# Supplementary material for: Age-related changes in the local milieu of inflamed tissues cause aberrant neutrophil trafficking and subsequent remote organ damage
Source: Immunity. 2021 Jul 13;54(7):1494–1510.e7. doi: 10.1016/j.immuni.2021.04.025 (PMC8284598; doi:10.1016/j.immuni.2021.04.025)
Supplement: Document S1. Figures S1–S7 [file mmc1.pdf]

Immunity, Volume *54*

Supplemental information

Age-related changes in the local milieu of inflamed tissues cause aberrant neutrophil trafficking and subsequent remote organ damage

Anna Barkaway, Loïc Rolas, Régis Joulia, Jennifer Bodkin, Tchern Lenn, Charlotte Owen-Woods, Natalia Reglero-Real, Monja Stein, Laura Vázquez-Martínez, Tamara Girbl, Robin N. Poston, Matthew Golding, Rebecca S. Saleeb, Aude Thiriot, Ulrich H. von Andrian, Johan Duchene, Mathieu-Benoit Voisin, Cleo L. Bishop, David Voehringer, Axel Roers, Antal Rot, Tim Lämmermann, and Sussan Nourshargh

Figure S1

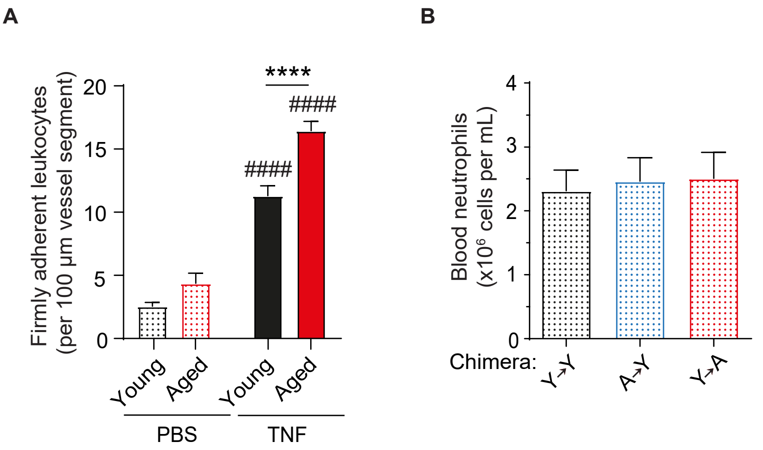

Figure S1 (Related to Figure 1). Leukocyte adhesion responses in TNF-stimulated venules of WT mice and chimeric mice characterization. (**A**) Young (2-4 months) and aged (≥18 months) mice were treated intrascrotally (i.s.) with PBS or TNF and leukocyte firm adhesion in cremasteric post-capillary venules quantified by brightfield IVM (n=3-7 mice/group). (**B**) Total neutrophil blood counts in chimeric mice (as generated in Figure 1H) at 4 weeks post irradiation and bone marrow transfer (n≥10 mice/group). Means ± SEM, ####p< 0.0001 as compared to age-matched controls, ****p<0.0001 as indicated.

Figure S2

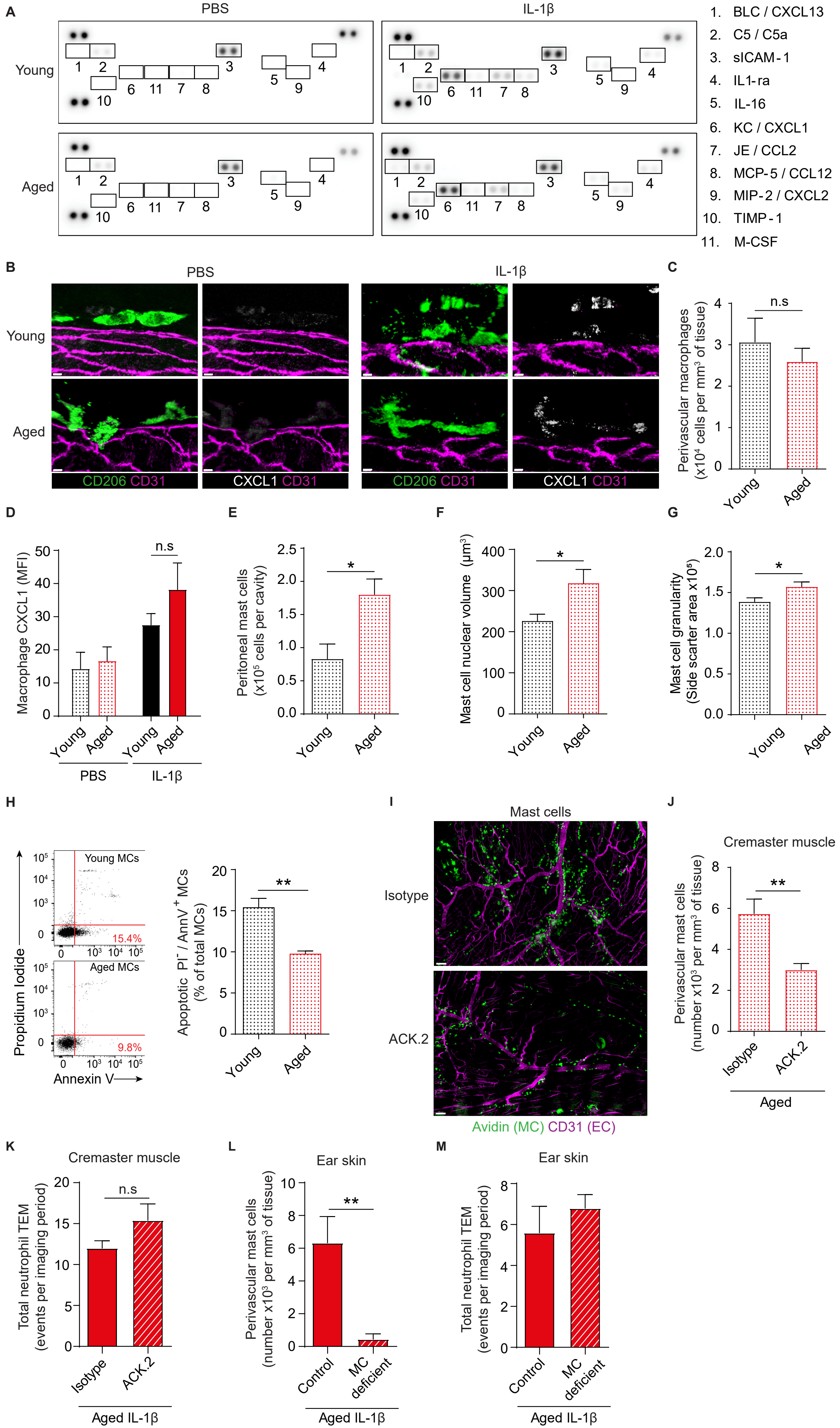
A
PBS
IL-1β
Young
Aged
1. BLC / CXCL13
2. C5 / C5a
3. sICAM-1
4. IL1-ra
5. IL-16
6. KC / CXCL1
7. JE / CCL2
8. MCP-5 / CCL12
9. MIP-2 / CXCL2
10. TIMP-1
11. M-CSF
B
CD206 CD31
CXCL1 CD31
C
Perivascular macrophages (x10⁴ cells per mm³ of tissue)
n.s
D
Macrophage CXCL1 (MFI)
E
Peritoneal mast cells (x10⁵ cells per cavity)
F
Mast cell nuclear volume (µm³)
G
Mast cell granularity (Side scarter area x10⁵)
H
Young MCs
Aged MCs
15.4%
9.8%
Propidium Iodide
Annexin V
Apoptotic PI⁻ / AnnV⁺ MCs (% of total MCs)
I
Mast cells
Isotype
ACK.2
Avidin (MC) CD31 (EC)
J
Cremaster muscle
Perivascular mast cells (number x10³ per mm³ of tissue)
K
Total neutrophil TEM (events per imaging period)
Aged IL-1β
L
Ear skin
Control
MC deficient
M

Figure S2 (Related to Figure 2). Characterization of pro-inflammatory mediator generation and stromal cells in young and aged stimulated cremasteric tissues. (**A**-**D**) Young and aged WT mice were stimulated i.s. with IL-1β or PBS for 4 h. (**A**) Inflammatory mediator detection by protein array in cremaster muscles (n=3 mice/condition). (**B**) Representative confocal images depicting CXCL1 expression by perivascular macrophages (CD206; scale bar: 5 μm), (**C**) macrophage numbers associated with post-capillary venules and (**D**) immunoreactive CXCL1 expression (MFI) in these cells (n=4-6 mice/group). (**E**) Number of peritoneal mast cells (MCs) in naïve young and aged WT mice as assessed by flow cytometry (n=3-5 mice/group). (**F**) Quantification of MC nuclear volume in un-stimulated ear skins as assessed by confocal microscopy (n=3 mice/group). Peritoneal MCs acquired from un-stimulated young and aged mice assayed for (**G**) granularity (n=5 mice/group) and (**H**) apoptosis (n=6-7 mice/group), both by flow cytometry. **(I-K)** Aged WT mice were treated with anti-CD117 (ACK.2) mAb or isotype control for mast cell (MC) depletion. (**I**) Representative tile scan confocal images of the cremaster muscle microcirculation (scale bar: 300 μm), (**J**) quantification of MCs (avidin) associated with post-capillary venules (CD31) (n=3-5 mice/group) and (**K**) total neutrophil TEM events in IL-1β-stimulated control or mast cell depleted aged mice. (**L**) Quantification of MC numbers associated with post-capillary venules in ear skins stained with avidin (MCs) and anti-CD31 mAb (ECs) and (**M**) total neutrophil TEM events in IL-1β-stimulated ear skin of MC deficient (*Mcpt5-Cre-R-DTA)* aged mice and their littermate controls (n=5-7 mice/group). Means ± SEM, *p<0.05, **p<0.01, n.s. not significant as indicated.

Figure S3

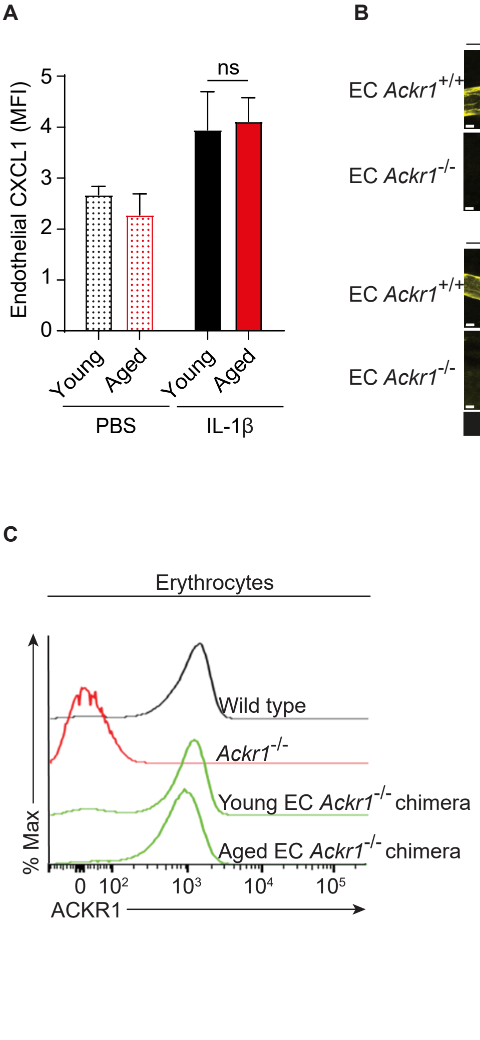

Figure S3 (Related to Figure 3) EC CXCL1 expression and EC *Ackr1*$^{-/-}$ chimeric mice characterization. (**A**) EC CXCL1 quantification (MFI) in young and aged mouse cremasteric post capillary venules (PCVs) treated with PBS or IL-1β (n = 6-7 mice/group). (**B**) Representative confocal images of ear skin PCVs immunostained for ACKR1 and CD31 from young and aged EC *Ackr1*$^{+/+}$ and EC *Ackr1*$^{-/-}$ chimeric mice (scale bar: 20 µm). (**C**) Flow cytometry analysis of erythrocyte (Ter119^{+}) ACKR1 expression and **(D)** relative percentages of ACKR1^{+} and ACKR1^{-} erythrocytes in peripheral blood of chimeric mice generated as described in Figure 3H (n=3-4 mice/group). Means ± SEM, n.s. not significant as indicated.

Figure S4

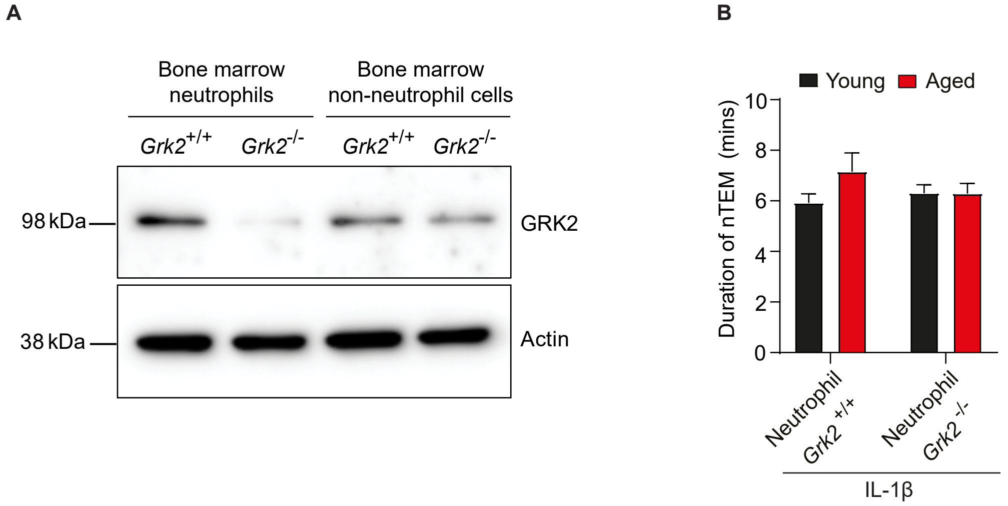

Figure S4 (Related to Figure 4). Validation of neutrophil GRK2 deficiency and analysis of neutrophil TEM duration. (**A**) GRK2 expression in purified bone marrow derived neutrophils and non-neutrophil cells of neutrophil *Grk2*$^{+/+}$ and *Grk*2$^{-/-}$ chimeric mice as analyzed by western blot (loading control: Actin). (**B**) Duration of neutrophil normal TEM (mins) as assessed by confocal IVM in IL-1β-stimulated cremaster muscles of young and aged neutrophil *Grk2*$^{+/+}$ and *Grk*2$^{-/-}$ chimeric mice (n=30-82 neutrophils analyzed/group). Means ± SEM.

Figure S5

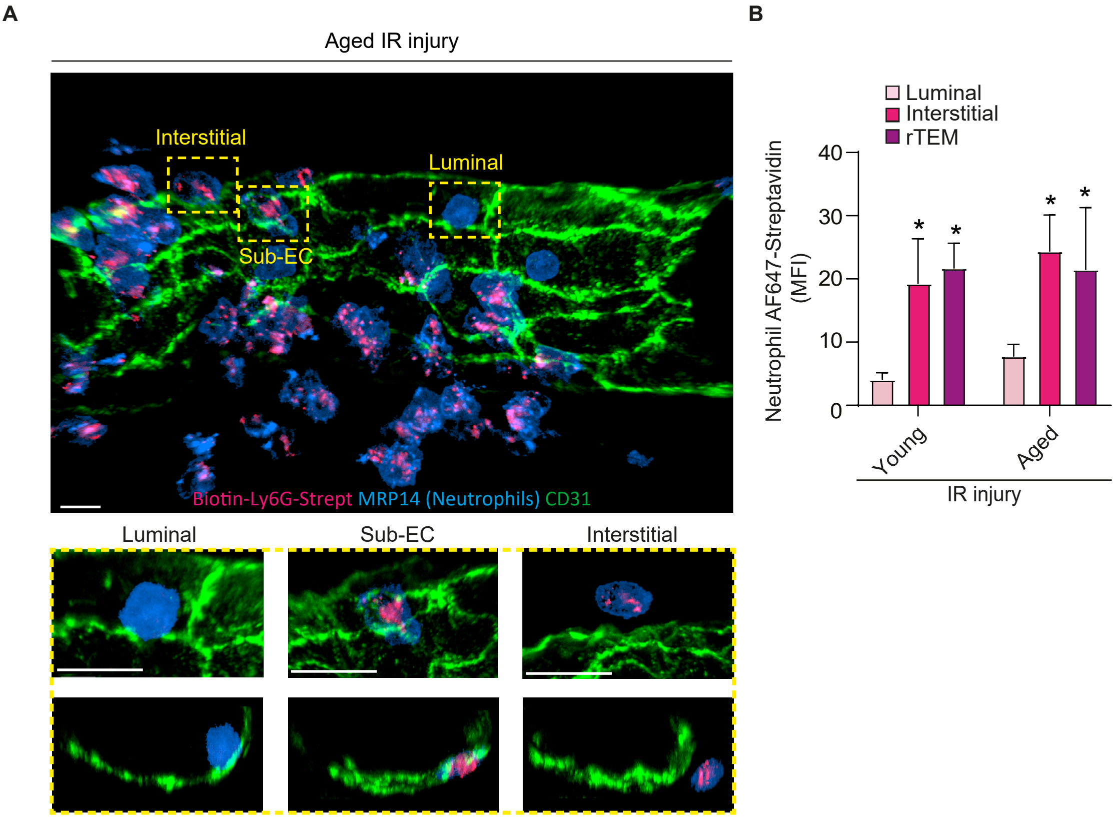

Figure S5 (Related to Figure 5). Validation of biotin-Ly6G-AF647-Streptavidin cell tracking, as a method for specifically labelling neutrophils that have breached the endothelium. Young and aged WT mice were injected i.v. with biotinylated anti-Ly6G mAb, subjected to sham or cremasteric IR injury and AF647-Strept was applied locally to cremaster muscles. (**A**) Representative confocal images of neutrophil AF647-Strept signal in the vascular lumen, subendothelial space and interstitium during IR injury in aged WT mice (dashed boxes delineate magnified areas; scale bar: 10 μm). (**B**) AF647-Strept quantification (MFI) of neutrophils within the lumen, interstitial tissue, and cells undergoing rTEM in IR-stimulated cremaster muscles of young and aged *Lyz2-EGFP-ki* mice (n=3-4 mice/group). Means ± SEM * $p<0.05$ as compared to MFI of age-matched luminal neutrophils.

Figure S6

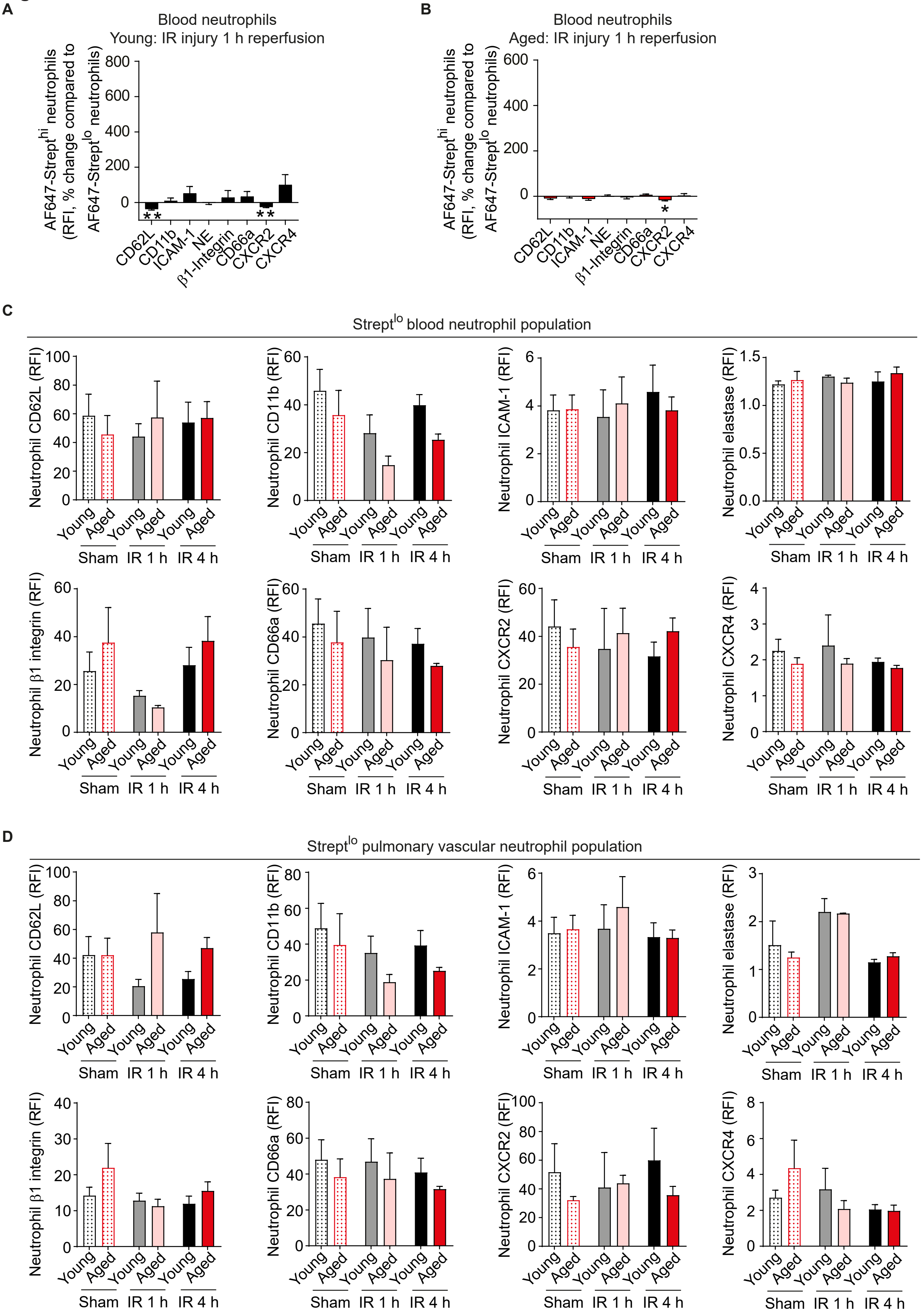
A
Blood neutrophils
Young: IR injury 1 h reperfusion
AF647-Strept^hi neutrophils (RFI, % change compared to AF647-Strept^lo neutrophils)
CD62L
CD11b
ICAM-1
NE
β1-integrin
CD66a
CXCR2
CXCR4
B
Blood neutrophils
Aged: IR injury 1 h reperfusion
C
Strept^lo blood neutrophil population
Neutrophil CD62L (RFI)
Neutrophil CD11b (RFI)
Neutrophil ICAM-1 (RFI)
Neutrophil elastase (RFI)
Neutrophil β1 integrin (RFI)
Neutrophil CD66a (RFI)
Neutrophil CXCR2 (RFI)
Neutrophil CXCR4 (RFI)
Young
Aged
Sham
IR 1 h
IR 4 h
D
Strept^lo pulmonary vascular neutrophil population

Figure S6 (Related to Figure 6). Phenotypic analysis of AF647-Strept^{lo} and AF647-Strept^{hi} blood and pulmonary vascular neutrophils. Young and aged WT mice were injected i.v. with biotinylated anti-Ly6G mAb, subjected to sham or cremasteric IR injury and AF647-Strept was applied locally to cremaster muscles. Relative expression levels of indicated markers on AF647-Strept^{hi} neutrophils relative to levels on AF647-Strept^{lo} neutrophils in the blood following 1 h reperfusion in **(A)** young and **(B)** aged IR-stimulated mice. Relative expression of indicated markers on AF647-Strept^{lo} (**C**) blood neutrophils and (**D**) neutrophils from the pulmonary vasculature (n=5 mice/group). Means ± SEM *p<0.05, **p<0.01 as compared to AF647-Strept^{lo} neutrophils of the same group.

Figure S7

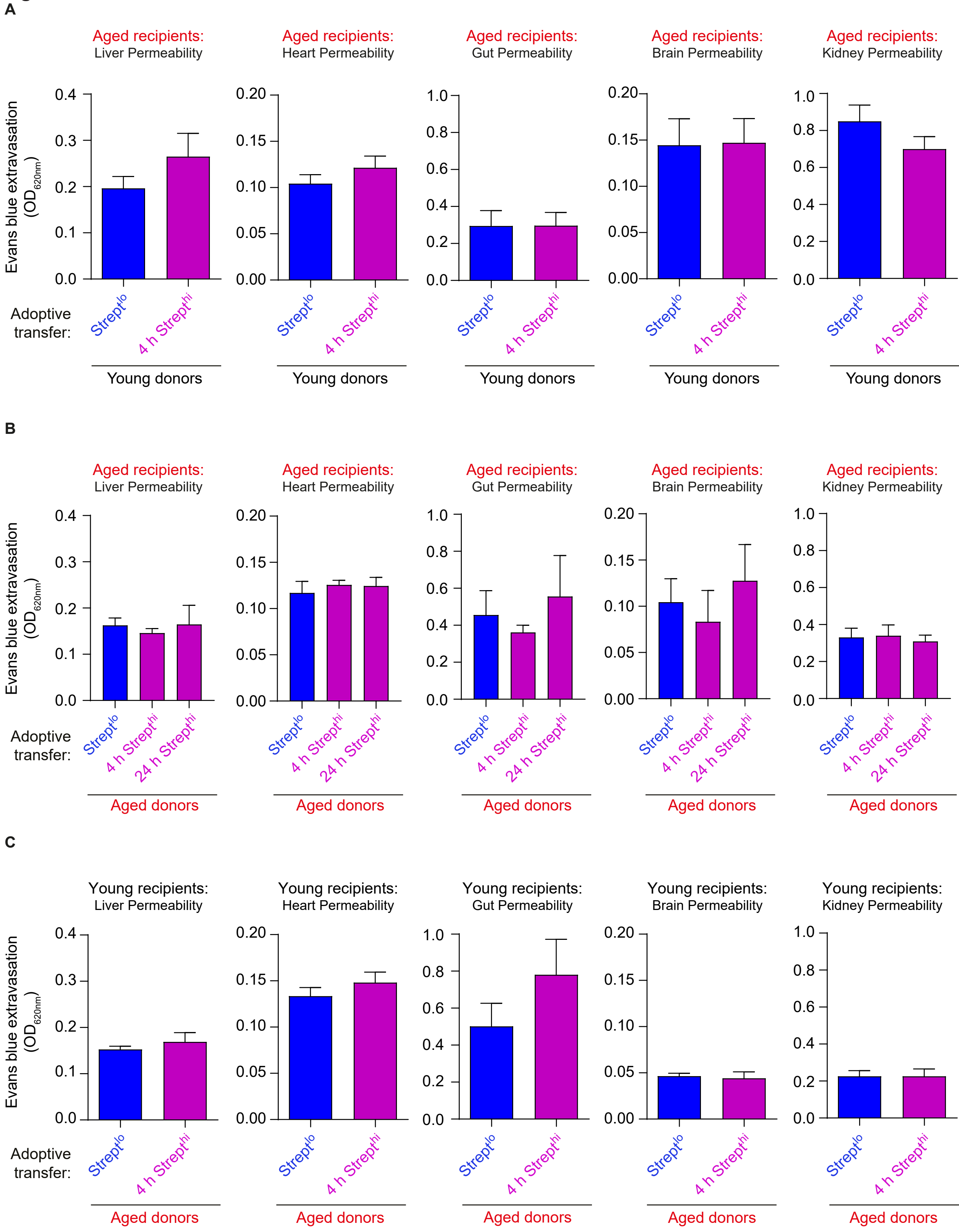

Figure S7 (Related to Figure 6). Impact of AF647-Strept^{lo} and AF647-Strept^{hi} neutrophils on organ permeability. Extravasation of i.v. Evans blue in multiple tissues of (**A**) aged recipients 4 h post i.v. injection of neutrophils sorted from young donors, (**B**) aged recipients 4 or 24 h post i.v. injection of neutrophils sorted from aged donors, and (**C**) young recipients 4 h post i.v. injection of neutrophils sorted from aged donors (n=4-7 mice/group). Means ± SEM.
