# Supplementary material for: Age-related changes in the local milieu of inflamed tissues cause aberrant neutrophil trafficking and subsequent remote organ damage
Source: Immunity. 2021 Jul 13;54(7):1494–1510.e7. doi: 10.1016/j.immuni.2021.04.025 (PMC8284598; doi:10.1016/j.immuni.2021.04.025)
Supplement: Document S2. Article plus supplemental information [file mmc7.pdf]

Article

Immunity

Age-related changes in the local milieu of inflamed tissues cause aberrant neutrophil trafficking and subsequent remote organ damage

Graphical abstract

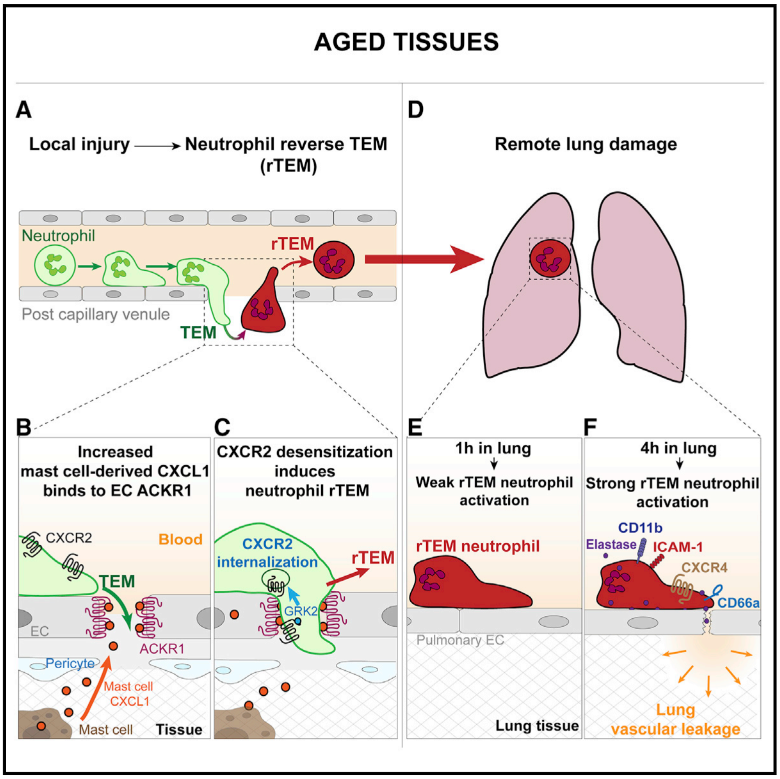

Authors

Anna Barkaway, Loïc Rolas, Régis Joulia, ..., Antal Rot, Tim Lämmermann, Sussan Nourshargh

Correspondence

s.nourshargh@qmul.ac.uk

In brief

Aging is a critical risk factor for inflammatory disorders. Barkaway, Rolas et al. show that inflamed aged tissues present a high frequency of neutrophil reverse transendothelial migration (rTEM) back into the circulation in a mast cell-dependent manner. rTEM neutrophils are retained in aged lungs and programmed toward an activated phenotype, capable of inducing tissue damage.

Highlights

- Aged mice show high levels of neutrophil reverse transendothelial migration (rTEM)
- Mast cells (MC) and MC-derived CXCL1 drive neutrophil rTEM in inflamed aged tissues
- Intensified endothelial ACKR1-CXCL1 axis promotes neutrophil CXCR2 internalization
- Aged lungs program rTEM neutrophils toward an activated and noxious phenotype

Barkaway et al., 2021, Immunity *54*, 1494–1510
July 13, 2021 © 2021 The Author(s). Published by Elsevier Inc.
https://doi.org/10.1016/j.immuni.2021.04.025

CellPress

CellPress
OPEN ACCESS

Immunity

Article

Age-related changes in the local milieu of inflamed tissues cause aberrant neutrophil trafficking and subsequent remote organ damage

Anna Barkaway,[1,11] Loïc Rolas,[1,11] Régis Joulia,[1] Jennifer Bodkin,[1] Tchern Lenn,[1] Charlotte Owen-Woods,[1] Natalia Reglero-Real,[1] Monja Stein,[1] Laura Vázquez-Martínez,[1] Tamara Girbl,[1] Robin N. Poston,[1] Matthew Golding,[1] Rebecca S. Saleeb,[1] Aude Thiriot,[2,3] Ulrich H. von Andrian,[2,3] Johan Duchene,[4] Mathieu-Benoit Voisin,[1] Cleo L. Bishop,[5] David Voehringer,[6] Axel Roers,[7] Antal Rot,[1,8] Tim Lämmermann,[9] and Sussan Nourshargh[1,8,10,*]

[1]Centre for Microvascular Research, William Harvey Research Institute, Barts and The London School of Medicine and Dentistry, Queen Mary University of London, London EC1M 6BQ, UK
[2]Department of Immunology and HMS Center for Immune Imaging, Harvard Medical School, Boston, MA, MA 02115, USA
[3]The Ragon Institute of MGH, MIT and Harvard, Cambridge MA 02139, USA
[4]Institute for Cardiovascular Prevention (IPEK), Ludwig-Maximillians-Universität (LMU) München, Munich 80336, Germany
[5]Centre for Cell Biology and Cutaneous Research, Blizard Institute, Barts and The London School of Medicine and Dentistry, Queen Mary University of London, London E1 2AT, UK
[6]Department of Infection Biology, University Hospital Erlangen and Friedrich-Alexander University Erlangen-Nuremberg (FAU), Erlangen 91054, Germany
[7]Institute for Immunology, Medical Faculty Carl Gustav Carus, Technische Universität Dresden, Dresden 01069, Germany
[8]Centre for Inflammation and Therapeutic Innovation, Barts and The London School of Medicine and Dentistry, Queen Mary University of London, London EC1M 6BQ, UK
[9]Max Planck Institute of Immunobiology and Epigenetics, Freiburg, Germany
[10]Lead contact
[11]These authors contributed equally
*Correspondence: s.nourshargh@qmul.ac.uk
https://doi.org/10.1016/j.immuni.2021.04.025

SUMMARY

Aging is associated with dysregulated immune functions. Here, we investigated the impact of age on neutrophil diapedesis. Using confocal intravital microscopy, we found that in aged mice, neutrophils adhered to vascular endothelium in inflamed tissues but exhibited a high frequency of reverse transendothelial migration (rTEM). This retrograde breaching of the endothelium by neutrophils was governed by enhanced production of the chemokine CXCL1 from mast cells that localized at endothelial cell (EC) junctions. Increased EC expression of the atypical chemokine receptor 1 (ACKR1) supported this pro-inflammatory milieu in aged venules. Accumulation of CXCL1 caused desensitization of the chemokine receptor CXCR2 on neutrophils and loss of neutrophil directional motility within EC junctions. Fluorescent tracking revealed that in aged mice, neutrophils undergoing rTEM re-entered the circulation and disseminated to the lungs where they caused vascular leakage. Thus, neutrophils stemming from a local inflammatory site contribute to remote organ damage, with implication to the dysregulated systemic inflammation associated with aging.

INTRODUCTION

Aging is a high-risk factor for the onset of inflammatory conditions, especially in life-threatening pulmonary and cardiovascular disorders (Akbar and Gilroy, 2020; Ferrucci and Fabbri, 2018; Nikolich-Žugich, 2018; Robba et al., 2020; Vabret et al., 2020). Regardless of the primary insult being a pathogenic microbe or sterile injury, a significant cause of mortality and comorbidity in older patients is increased susceptibility to organ dysfunction remote from the initial inflammatory trigger. This is illustrated by the SARS-CoV-2 pandemic, as elderly patients with COVID-19 are particularly at risk of pneumonia but also present multiple organ failure (Akbar and Gilroy, 2020; Robba et al., 2020; Vabret et al., 2020). Furthermore, damage to remote organs is a significant cause of intensive care admissions following physical trauma in older patients (Lord et al., 2014). Together, aging-associated remote organ dysfunction represents an important unmet clinical problem that requires greater mechanistic understanding.

Advanced age promotes immune dysregulation, a phenomenon considered to be a principal cause of aging-associated pathologies (Akbar and Gilroy, 2020; Nikolich-Žugich, 2018; Shaw et al., 2013). The underlying basis of this is complex and linked to multiple factors, such as compromised cell-intrinsic leukocyte

 © 2021 The Author(s). Published by Elsevier Inc.
This is an open access article under the CC BY license (http://creativecommons.org/licenses/by/4.0/).

CellPress
OPEN ACCESS

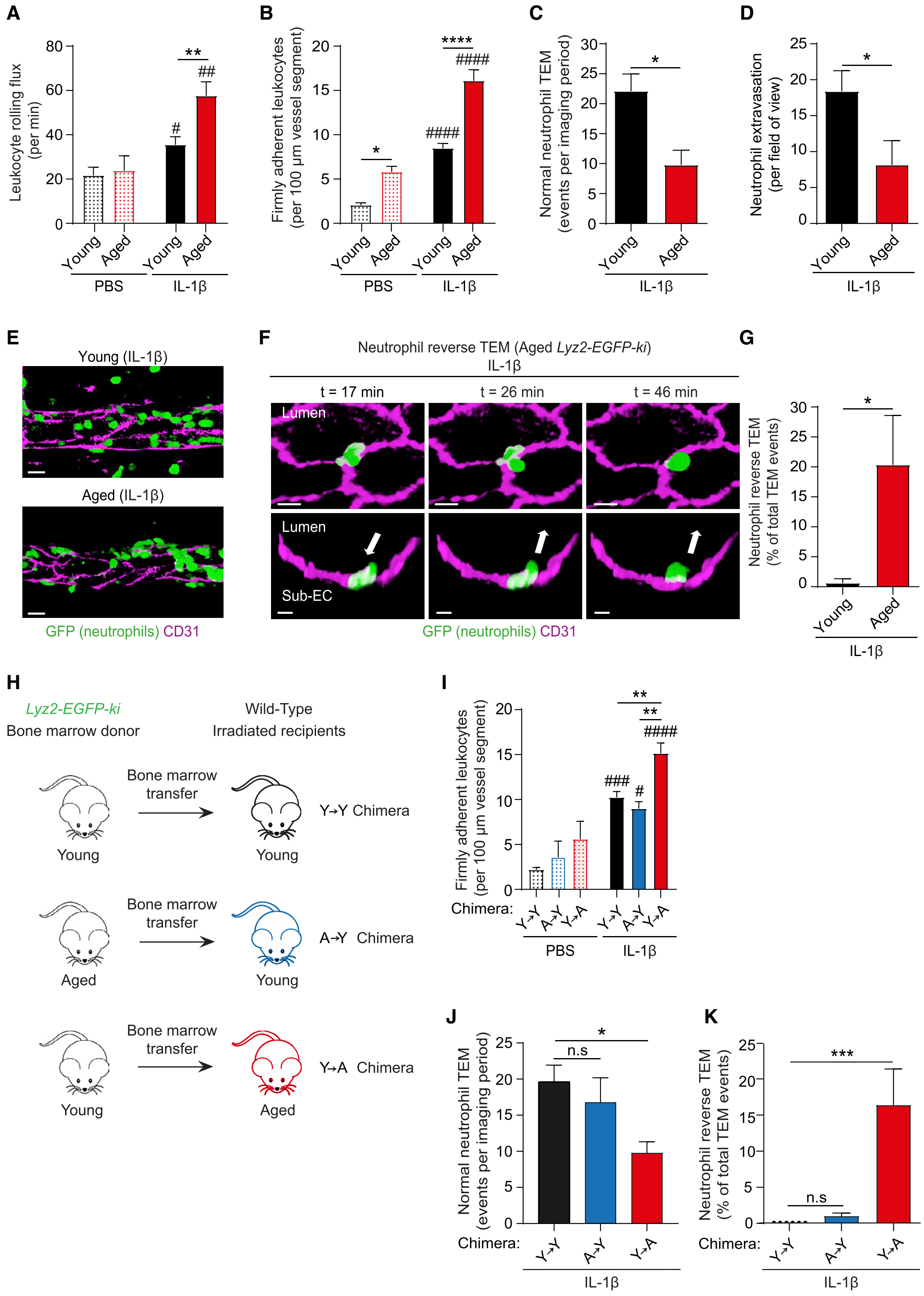

Figure 1. Inflamed aged stroma promotes aberrant neutrophil transendothelial cell migration

(A–G) Young (2–4 months) and aged (≥18 months) mice were treated intrascrotally (i.s.) with PBS or IL-1β and neutrophil responses in cremasteric post-capillary venules analyzed. Leukocyte (A) rolling flux and (B) firm adhesion in WT mice as quantified by brightfield IVM (n = 3–16 mice/group). Neutrophil (C) normal TEM

(legend continued on next page)

behaviors, changes in immune cell local microenvironments, and increased circulating pro-inflammatory mediators (Nikolich-Žugich, 2018; Shaw et al., 2013). While aging influences innate immunity, the associated reports are varied and many mechanistic questions remain (Nikolich-Žugich, 2018; Shaw et al., 2013). Neutrophil trafficking constitutes a crucial component of innate immunity and inflammatory disease states and studies of isolated neutrophils from aged individuals have revealed reduced chemotaxis *in vitro* (Fulop et al., 2004; Sapey et al., 2014). *In vivo*, dysregulated neutrophil trafficking in aged mice is aligned with factors such as aberrant production of systemic or local inflammatory mediators or diminished local anti-inflammatory mechanisms (Eskan et al., 2012; Gomez et al., 2007; Kulkarni et al., 2019; Nomellini et al., 2012; Nomellini et al., 2008; Wulfert et al., 2012).

To investigate the impact of age on the dynamics of neutrophil diapedesis, we examined neutrophil breaching of venular walls in inflamed tissues of aged mice in real-time using confocal intravital microscopy (IVM). We noted increased prevalence of transmigrating neutrophils exhibiting retrograde motility within endothelial cell (EC) junctions and re-entering the vascular lumen. Mechanistically, this neutrophil reverse transendothelial cell migration (rTEM) behavior was governed by the aged stroma (tissue) and mediated by elevated production of the chemokine CXCL1 by tissue resident mast cells. Increased expression of the atypical chemokine receptor 1 (ACKR1) in aged tissues facilitated the retention of CXCL1 within venular EC junctions which, in turn, induced downregulation of its cognate receptor, CXCR2, on transmigrating neutrophils. This resulted in loss of neutrophil directional motility with consequent neutrophil rTEM and re-entry of neutrophils back into the circulation. rTEM neutrophils were tracked from injured tissues to the lungs where, in aged mice, they were programmed toward an activated phenotype capable of causing tissue damage. Together, an intensified local pathway involving upregulation of mast cell-derived CXCL1 and EC ACKR1 was shown to mediate age-related changes in the local inflammatory milieu that is capable of prompting noxious neutrophil re-entry into the systemic circulation, ultimately inducing downstream remote organ injury.

RESULTS

Inflamed aged stroma promotes aberrant neutrophil transendothelial cell migration

The impact of age on neutrophil-venular wall interactions was investigated in inflamed mouse cremaster muscles that due to its translucency is amenable to high resolution IVM. Cremaster muscles were acutely inflamed via local injection of IL-1β in young and aged mice and leukocyte responses were investigated in real-time. Analysis of tissues of wild-type (WT) mice revealed significantly enhanced leukocyte rolling and adhesion in aged (≥18 months), as compared to young (2–4 months) mice (Figures 1A and 1B). A similar increase in leukocyte firm adhesion was observed in TNF-stimulated aged tissues (Figure S1A). Breaching of venular walls was investigated using neutrophil reporter mice *Lyz2-EGFP-ki* (display GFP^{bright} neutrophils) and following staining of EC junctions by locally administered non-blocking anti-CD31 mAb (Woodfin et al., 2011). Young *Lyz2-EGFP-ki* mice showed a notable frequency of neutrophil TEM events, typified by full breaching of the endothelium in a luminal-to-abluminal manner, a response termed normal TEM (nTEM; Video S1). Despite exhibiting increased neutrophil-EC adhesion (Figures 1A, 1B, and S1A), aged mice showed a lower number of neutrophil nTEM events (Figure 1C) and reduced neutrophil infiltration into the perivascular space (Figures 1D and 1E). These findings were accounted for by a high frequency of neutrophil retrograde motility within EC junctions (reverse TEM; rTEM) (~20% of all TEM events; Figures 1F and 1G; Video S2) that led to the eventual re-entry of the neutrophils back into the vascular lumen. In line with our previous findings (Owen-Woods et al., 2020; Woodfin et al., 2011), IL-1β-inflamed tissues of young mice showed negligible evidence of neutrophil rTEM (Figure 1G). We also observed neutrophil rTEM in IL-1β-stimulated ear skin of aged mice (Video S3), a model that exhibited comparable levels of neutrophil rTEM in male and female animals (~12% in relation to all TEM events).

To investigate the relative contribution of neutrophils versus the stroma (tissue) to the observed dysregulated neutrophil TEM in aged mice, a series of bone marrow (BM) chimeric animals were established (Figure 1H). Two chimeric cohorts were generated by BM transfer from aged and young *Lyz2-EGFP-ki* mice into irradiated young and aged WTs, respectively. This yielded animals with aged hematopoietic cells and young stroma (A→Y), and conversely, mice with young hematopoietic cells and aged stroma (Y→A). A control group of young irradiated WT mice received BM from young *Lyz2-EGFP-ki* mice (Y→Y). All chimeras exhibited similar blood neutrophil counts (Figure S1B) and reconstitution levels of GFP^{bright} neutrophils (≥95%). Aged chimeras showed similar vascular, stromal, and functional characteristics as compared to non-irradiated aged mice (Table S1). While all chimeric mice displayed robust luminal adhesion, this was increased in Y→A chimeras (exhibiting aged stroma) as compared to A→Y and Y→Y chimeras (Figure 1I). However, Y→A chimeras displayed reduced nTEM (Figure 1J) and reduced neutrophil migration into the perivascular tissue (~43%), as compared to Y→Y control chimeric animals. Furthermore, although almost absent in Y→Y and A→Y chimeras, Y→A chimeras exhibited an increased frequency of

events (n = 5-7 mice/group; Video S1), (D) total extravasation (n = 5–7 mice/group), and (E) related representative images of *Lyz2-EGFP-ki* venules, as assessed by confocal IVM (scale bar: 15 μm).

(F) Time-lapse confocal images (Video S2) showing a neutrophil rTEM event in an IL-1β-stimulated aged *Lyz2-EGFP-ki* venule with the neutrophil in the subendothelial space (t = 17 min) re-entering the vascular lumen (t = 26 min to t = 46 min). Top panel: *en face* luminal view; bottom panel: cross-sections; arrows: direction of neutrophil motility (scale bar: 10 μm).

(G) Frequency of neutrophil rTEM in *Lyz2-EGFP-ki* stimulated tissues (n = 5–6 mice/group).

(H) The generation of Y→Y, A→Y, or Y→A chimeras (young 'Y'; or aged 'A') and (I–K) their analysis post treatment with i.s. PBS or IL-1β. Cremaster muscle (I) leukocyte firm adhesion as assessed by brightfield IVM (n = 3-10 mice/group), (J) neutrophil normal TEM events (n = 4-5 mice/group) and (K) frequency of neutrophil rTEM as assessed by confocal IVM (n = 3-5 mice/group). Means ± SEM, #p < 0.05, ##p < 0.01, ###p < 0.001, ####p < 0.0001 relative to aged-matched controls and *p < 0.05, **p < 0.01, ***p < 0.001, ****p < 0.0001, n.s. not significant, as indicated.

See also Figure S1.

CellPress
OPEN ACCESS

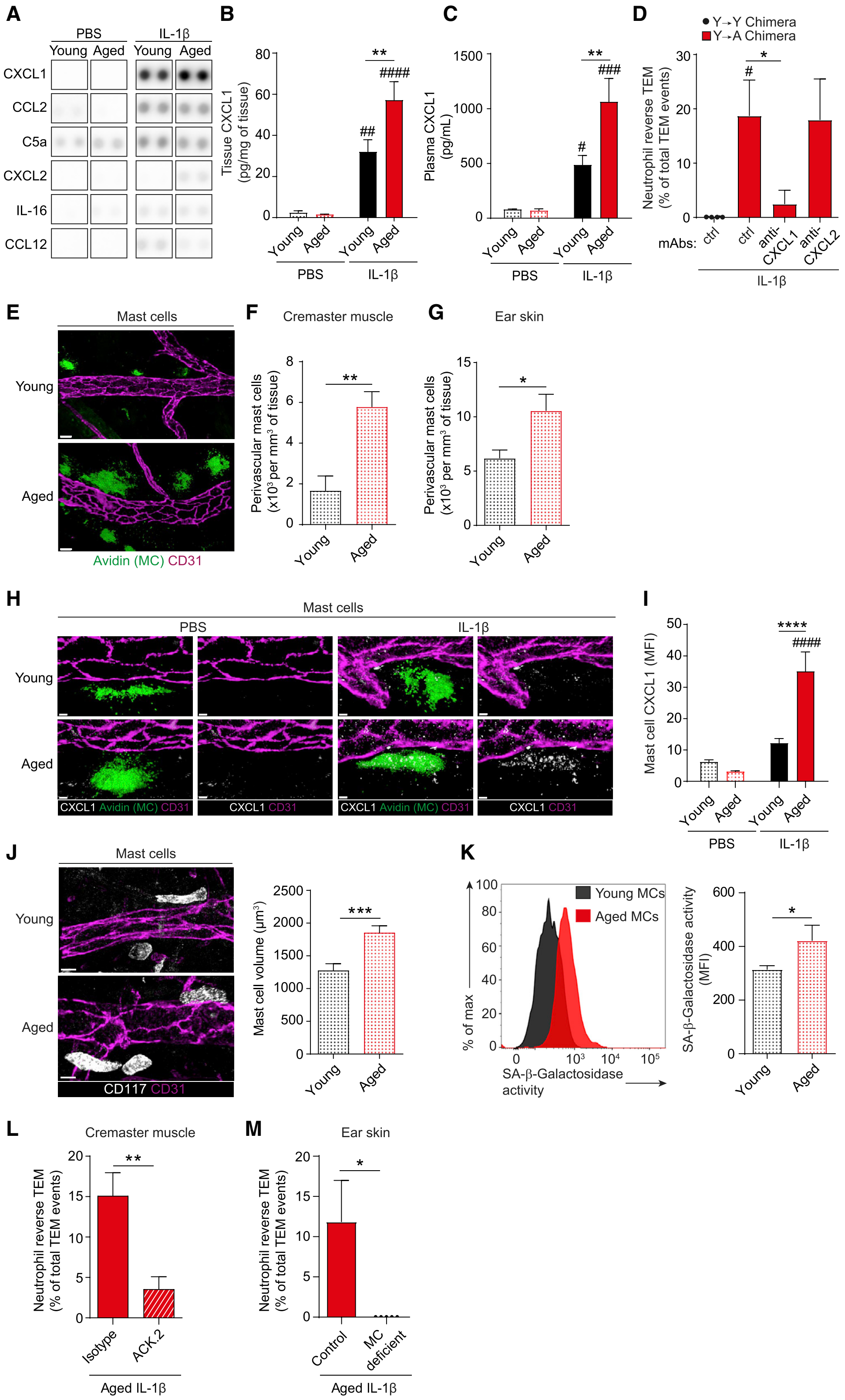

(legend on next page)

neutrophil rTEM (~15%; Figure 1K). In line with these findings, GFP^{bright} BM neutrophils of young donors adoptively transferred into IL-1β-stimulated WT mice displayed 21.7% neutrophil rTEM in aged recipients, whereas no such events were seen in young recipients (0%; n = 4 mice/group). Collectively, aged mice exhibit disrupted neutrophil-EC interactions, characterized by a high frequency of neutrophil rTEM, with clear evidence of aged stroma driving this retrograde neutrophil migration.

CXCL1 drives aging-associated neutrophil reverse TEM

Since a heightened pro-inflammatory state is a feature of aging (Ferrucci and Fabbri, 2018; Nikolich-Žugich, 2018), we considered that aging-associated aberrant neutrophil TEM may be driven by a dysregulated local inflammatory milieu. To explore this, control (PBS) and IL-1β-stimulated tissues of young and aged mice were analyzed by a Cytokine Array and ELISA. IL-1β-stimulated tissues produced increased levels of numerous pro-inflammatory mediators (Figures 2A and S2A). Among these, the chemokine CXCL1, a potent neutrophil chemoattractant, was significantly increased in IL-1β-stimulated aged as compared to young tissues, a profile also observed in plasma (Figures 2B and 2C).

Hypothesizing that high levels of CXCL1 in inflamed aged tissues might cause the observed increase in neutrophil rTEM, IL-1β-stimulated chimeric mice with aged stroma (Y→A) were treated with blocking anti-CXCL1 mAb or an isotype control and analyzed by confocal IVM. Anti-CXCL1 mAb treatment had no significant effect on total neutrophil TEM events (12.8 ± 2.3 as compared with 13.5 ± 3.8 after isotype, n = 4–5 mice/group), but significantly suppressed the frequency of neutrophil rTEM (~89% inhibition; Figure 2D). In contrast, an anti-CXCL2 mAb had no inhibitory effect on the frequency of neutrophil rTEM in this inflammatory model (Figure 2D).

Next, to shed light on the mechanisms in aging-associated overproduction of CXCL1, we investigated its cellular sources. Here, we focused on tissue resident macrophages and mast cells, known to be major sources of CXCL1 in early stages of neutrophil recruitment (De Filippo et al., 2013). Immunofluorescence (IF) staining of control and IL-1β-stimulated cremaster muscles showed no significant difference in macrophage numbers (Figure S2C) or macrophage-associated CXCL1 in perivascular regions of young and aged tissues (Figures S2B and S2D). In contrast, numbers of mast cells were increased in aged tissues, compared to young (Figures 2E and 2F). This was consistent across multiple organs, with ear skin and peritoneal cavity of aged animals exhibiting increased mast cell numbers as compared to young tissues (Figures 2G and S2E). Furthermore, although PBS and IL-1β-stimulated cremaster muscles of young mice, and PBS-treated aged tissues, showed almost undetectable levels of mast cell-associated CXCL1, mast cells of inflamed aged tissues displayed ~187% per cell increase in protein expression of CXCL1, relative to stimulated young tissues (Figures 2H and 2I). Since mast cells of aged mice had a significantly greater cellular and nuclear volume (Figures 2J and S2F), were more granular (Figure S2G), and exhibited increased senescence-associated-β-galactosidase (SA-β-gal) activity (Figure 2K), their increased CXCL1 may be due to cellular senescence, i.e., the senescence-associated secretory phenotype (SASP) (Akbar and Gilroy, 2020; Nikolich-Žugich, 2018; Shaw et al., 2013). Furthermore, mast cells residing in aged tissues displayed reduced apoptosis (Figure S2H), suggesting their high number may represent increased survival.

Having identified mast cells as a key cellular source of enhanced tissue-derived CXCL1, we next directly investigated their role in age-related neutrophil rTEM. We initially analyzed aged chimeric mice depleted of their cremasteric mast cells by local administration of an anti-c-kit (ACK.2) mAb (Brandt et al., 2003), which led to ~48% depletion of perivascular mast cells (Figures S2I and S2J). This treatment had no impact on the total number of TEM events (Figure S2K) but significantly suppressed the frequency of neutrophil rTEM within cremasteric venules (Figure 2L) as compared to control mice. Similarly, aged mast cell deficient mice (*Mcpt5-Cre-R-DTA*; Figure S2L) showed comparable levels of neutrophil TEM to their aged-matched littermate controls (Figure S2M), but no neutrophil rTEM in IL-1β–stimulated ear skin (Figure 2M). Together, the results identify mast cells as a key cellular source of tissue-derived CXCL1 and a major driver of neutrophil rTEM in acutely inflamed aged tissues.

ACKR1 is elevated in aged tissues and retains mast cell-derived CXCL1 at EC junctions

The dynamics and directionality of neutrophil TEM is exquisitely regulated by chemokines that are locally generated and strategically presented to migrating leukocytes (Girbl et al., 2018; Nourshargh and Alon, 2014). In line with this fundamental concept, we hypothesized that excessive CXCL1 in inflamed

Figure 2. CXCL1 drives aging-associated neutrophil reverse TEM

(A–D) Young and aged mice were treated i.s. with PBS or IL-1β. (A) Inflammatory mediator analysis in homogenized cremaster muscles as assayed by protein array (n = 3 mice/condition).

(B) CXCL1 levels in cremaster muscles (n = 4-7 mice/group) or (C) plasma (n = 4–8 mice/group) as quantified by ELISA.

(D) Frequency of neutrophil rTEM in Y→Y or Y→A chimeras (generated as detailed in Figure 1H) treated i.v. with isotype control, anti-CXCL1 or anti-CXCL2 blocking mAbs (n = 3–5 mice/group).

(E) Representative confocal images of mast cells (MCs; Avidin) associated with post-capillary venules (CD31) in young and aged unstimulated WT cremaster muscles (scale bar: 20 μm) and quantification in (F) cremaster muscles, and (G) ear skin (n = 5-7 mice/group).

(H–I) Analysis of CXCL1 expression in MCs of young and aged IL-1β-stimulated cremasteric tissues by confocal microscopy with (H) showing representative images and (I) quantification by MFI (scale bar: 5 μm; n = 3–7 mice/group).

(J) Representative confocal images of MCs (CD117) in young and aged unstimulated WT ear skin (scale bar: 10 μm) and quantification of MC volume (n = 4 mice/group). (K) Peritoneal MCs acquired from unstimulated young and aged mice assayed for SA-β-galactosidase activity by flow cytometry (n = 6-13 mice/group). (L) Frequency of neutrophil rTEM in control and MC depleted IL-1β-stimulated cremaster muscles of aged chimeras (see Figure 1H; n = 4-5 mice/group). (M) Frequency of neutrophil rTEM in IL-1β-stimulated ear skin of aged MC deficient (*Mcpt5-Cre-R-DTA)* mice and littermate controls (n = 5 mice/group). Means ± SEM, #p < 0.05, ##p < 0.01, ###p < 0.001, ####p < 0.0001 relative to controls, *p < 0.05, **p < 0.01, ***p < 0.001, ****p < 0.0001 as indicated.

See also Figure S2.

CellPress
OPEN ACCESS

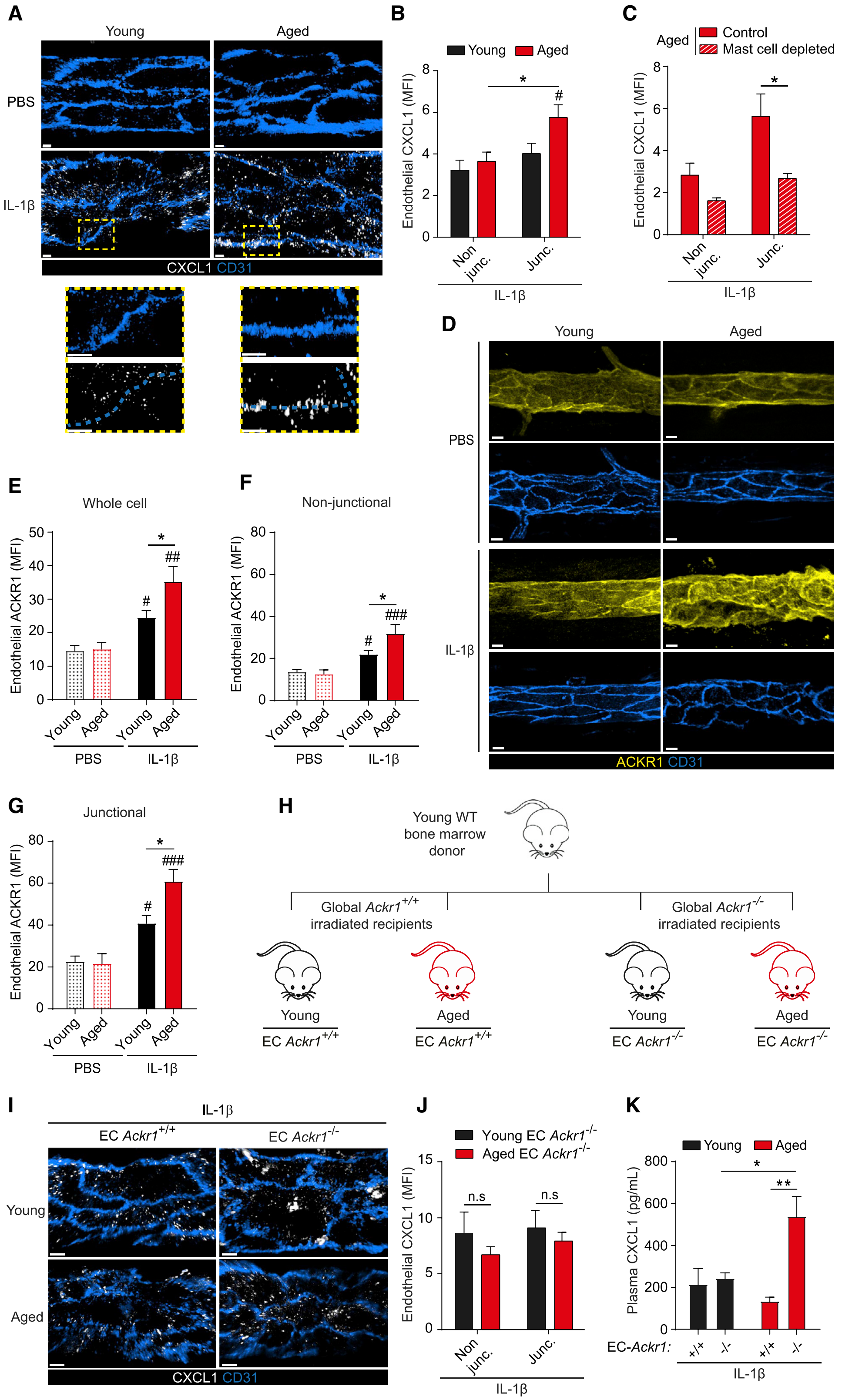

Figure 3. ACKR1 is elevated in aged tissues and retains mast cell-derived CXCL1 at EC junctions

(A–G) Young and aged WT mice were treated i.s. with PBS or IL-1β and cremaster muscles analyzed by confocal microscopy.

(A) Representative confocal images of post-capillary venules (PCVs) immunostained for CD31 and CXCL1 (scale bar: 4 μm; dashed boxes delineate magnified areas) and (B) quantification of CXCL1 expression (MFI) at EC junctional (junc.) and non-junctional (non-junc.) regions (n = 6-7 mice/group).

(legend continued on next page)

aged tissues may promote aberrant neutrophil TEM due to its altered patterning on venular ECs. To explore, cremaster muscles of young and aged mice were analyzed for CXCL1 localization by IF and confocal microscopy. Although PBS-treated tissues exhibited an almost undetectable CXCL1 signal on ECs, this was increased with IL-1β-treatment (Figures 3A and S3A). In stimulated young tissues, CXCL1 was evenly distributed in ECs but inflamed aged tissues exhibited increased junctional CXCL1 (Figures 3A and 3B). Aged mice depleted of their perivascular mast cells (Figures S2I and S2J) showed significantly reduced CXCL1 localization at EC junctions, comparable to levels detected in non-junctional regions (Figure 3C).

Aiming to elucidate the molecular basis of mast cell-derived CXCL1 retention at EC junctions, we focused on ACKR1. This atypical chemokine receptor binds CXCL1 and numerous other chemokines with high affinity (Novitzky-Basso and Rot, 2012) and is enriched at venular EC junctions (Girbl et al., 2018; Thiriot et al., 2017). While the overall EC expression of ACKR1 was increased in IL-1β-stimulated tissues, this response was most pronounced in aged mice (Figures 3D and 3E) that exhibited a distinct elevation in junctional localization of ACKR1 (Figures 3F and 3G). In addition to ECs, ACKR1 is expressed by erythroid cells in the BM and blood where it impacts chemokine homeostasis and neutrophil phenotypes (Duchene et al., 2017). To directly investigate the functional role of EC ACKR1 in retaining endogenously generated CXCL1 at EC junctions, we generated young and aged chimeras with selective EC ACKR1 deficiency (Figures 3H, S3B, S3C, and S3D). In analyzing CXCL1 expression, stimulated tissues of both young and aged EC *Ackr1*$^{-/-}$ chimeras showed characteristic punctate expression (Figure 3I). However, aged EC *Ackr1*$^{-/-}$ chimeras failed to show increased localization of CXCL1 at EC junctions (Figure 3J). While it is generally considered that erythrocyte ACKR1 governs the availability of plasma chemokines (Novitzky-Basso and Rot, 2012), we detected increased plasma levels of CXCL1 in locally stimulated aged EC *Ackr1*$^{-/-}$ chimeras as compared to aged EC *Ackr1*$^{+/+}$ mice (Figure 3K). These results suggest that EC ACKR1 can control circulating levels of chemokines produced by locally inflamed tissues. Collectively, inflamed aged tissues exhibit an aberrant vascular milieu characterized by an intensified expression of ACKR1 on venular ECs.

GRK2-dependent CXCR2 downregulation promotes neutrophil rTEM in aged tissues

Considering how increased retention of CXCL1 at EC junctions could influence neutrophil directional motility, we hypothesized that it might affect the expression of its cognate receptor CXCR2. IF staining showed CXCR2 expression on the plasma membrane of almost all luminal neutrophils in young tissues (Figure 4A). This was markedly reduced in aged mice, with ~30% of luminal neutrophils exhibiting low levels of membrane CXCR2 (Figures 4A). As these CXCR2^{lo} neutrophils were almost exclusively in close proximity to EC junctions (Figure 4A), we hypothesized that this phenotype may be caused by high levels of junctional CXCL1 retained by ACKR1. In addressing, we analyzed the frequency of CXCR2^{lo} neutrophils in stimulated venules of young and aged EC ACKR1 expressing and deficient chimeras. As found in aged animals (Figure 4A), aged chimeric EC *Ackr1*$^{+/+}$ mice showed an increased incidence of luminal CXCR2^{lo} neutrophils (Figure 4B). However, we detected few luminal CXCR2^{lo} neutrophils in aged EC *Ackr1*$^{-/-}$ chimeras (Figure 4B). These results indicate that, in inflamed aged tissues, high levels of EC junctional ACKR1, and increased levels of CXCL1 retained by it, promote downregulation of CXCR2 on transmigrating neutrophils.

To directly investigate the impact of reduced CXCR2 expression on neutrophil transmigration, we generated young and aged chimeric mice expressing or deficient in neutrophil G protein-coupled receptor kinase-2 (GRK2), one of a number of GRKs expressed by neutrophils known to regulate neutrophil GPCR signaling (Lämmermann and Kastenmüller, 2019). Irradiated young and aged WT recipients were injected i.v. with BM from mice with selective neutrophil GRK2 deficiency that were additionally intercrossed with *Lyz2-EGFP-ki* animals (*Mrp8-Cre;Grk2*$^{fl/fl}$*;Lyz2-EGFP-ki*; Figure 4C). Control chimeras were generated through use of *Grk2*$^{fl/fl}$*;Lyz2-EGFP-ki* mice as BM donors. Selective neutrophil GRK2 deficiency was confirmed using BM cells (Figure S4A) and all chimeras exhibited high levels of donor cell reconstitution in whole blood (≥95%). IL-1β-stimulated tissues of aged neutrophil *Grk2*$^{+/+}$ chimeras exhibited elevated levels of luminal CXCR2^{lo} neutrophils, as compared to young *Grk2*$^{+/+}$ chimeras, that was reduced in aged neutrophil *Grk2*$^{-/-}$ mice (Figure 4D). Functionally, neutrophil GRK2 deletion had no impact on total neutrophil TEM (Figure 4E) or its duration (Figure S4B) and stimulated young neutrophil *Grk2*$^{+/+}$ and young neutrophil *Grk2*$^{-/-}$ chimeras showed almost undetectable levels of neutrophil rTEM (Figure 4F). However, while aged chimeras harboring *Grk2*$^{+/+}$ neutrophils exhibited a marked frequency of neutrophil rTEM, this was reduced (~80% inhibition) in aged chimeras expressing *Grk2*$^{-/-}$ neutrophils. These results provide evidence that GRK2-mediated CXCR2 internalization induces loss of neutrophil directional motility within EC junctions of aged tissues. Collectively, in aged mice, excessive EC junctional CXCL1 elicits neutrophil CXCR2 internalization, prompting neutrophils that had initiated diapedesis to re-enter the vascular lumen.

rTEM neutrophils stemming from locally injured aged tissues accumulate in lungs

To assess the dispersion and systemic pathophysiological impact of rTEM neutrophils in aged mice, we extended our investigations to a model of local ischemia-reperfusion (IR) injury. This inflammatory insult, a hallmark of numerous aging-associated pathologies,

(C) EC CXCL1 expression (MFI) in control and mast cell-depleted aged cremaster tissues (n = 3-5 mice/group). (D) Representative confocal images illustrating ACKR1 expression in PCVs (CD31; scale bar: 10 μm) and ACKR1 quantification (MFI) within (E) whole ECs, and EC (F) non-junctional or (G) junctional regions (n = 3 mice/group).
(H) Generation of EC *Ackr1*$^{+/+}$ and EC *Ackr1*$^{-/-}$ chimeras. (I-K) Young and aged chimeras as generated in (H) were treated i.s. with IL-1β.
(I) Representative confocal images of cremasteric PCVs immunostained for CD31 and CXCL1 (scale bar: 4 μm), (J) quantification of CXCL1 expression (MFI) within EC junctional and non-junctional regions (n = 3-8 mice/group) and (K) plasma CXCL1 as quantified by ELISA (n = 3-8 mice/group). Means ± SEM, #p < 0.05, ##p < 0.01, ###p < 0.001 relative to controls, *p < 0.05, **p < 0.01, n.s. not significant, as indicated.
See also Figure S3.

CellPress
OPEN ACCESS

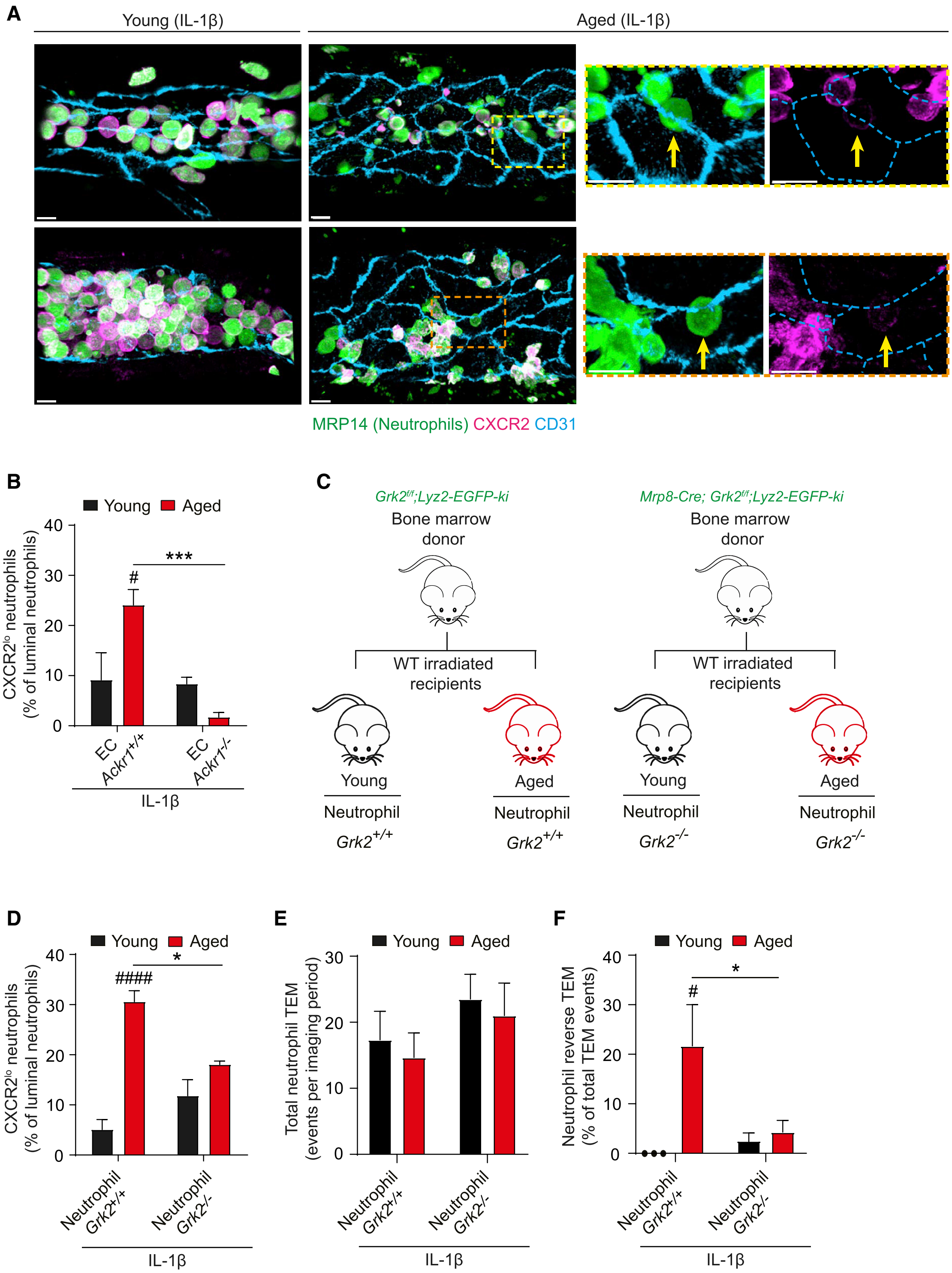

Figure 4. GRK2-dependent CXCR2 downregulation promotes neutrophil rTEM in aged tissues.

(A–F) Young and aged mice were treated i.s. with IL-1β.

(A) Representative confocal images of cremasteric post-capillary venules (PCVs) of WT mice immunostained for CXCR2, MRP14 (neutrophils) and CD31. Arrows indicate CXCR2^{lo} neutrophils (scale bar: 10 μm; dashed boxes delineate magnified areas).

(B) Percentage of luminal CXCR2^{lo} neutrophils in cremasteric PCVs of EC *Ackr1*$^{+/+}$ and EC *Ackr1*$^{-/-}$ chimeras (n = 3-5 mice/group).

(C) Generation of neutrophil *Grk2*$^{+/+}$ and *Grk2*$^{-/-}$ chimeras.

(D–F) Young and aged chimeras as generated in (C) were treated i.s. with IL-1β.

(legend continued on next page)

elicited an intense neutrophil infiltration in both young and aged WT mice (Figure 5A). Since aging is associated with greater susceptibility to remote organ failure, we analyzed mice subjected to this local IR model for lung injury. Here, as indicated by increased extravascular accumulation of i.v. administered fluorescent beads (20 nm), aged mice exhibited significantly greater lung damage as compared to young at 4 h post reperfusion (Figure 5B). This response appeared to be sustained, in that notable lung permeability was detected in aged mice even at 24 h post reperfusion (~40% increase as compared to young mice; n = 5 mice/group).

Since we have previously aligned neutrophil rTEM with distant organ damage (Colom et al., 2015; Owen-Woods et al., 2020; Woodfin et al., 2011), we next analyzed neutrophil TEM dynamics by confocal IVM. IR injury in mice expressing aged stroma (Y→A chimeras) was characterized by significant reduction in the number of nTEM events but increased frequency of neutrophil rTEM as compared to Y→Y chimeras (Figures 5C and 5D). Hypothesizing that the observed lung damage may be caused by greater frequency of rTEM neutrophils stemming from locally injured aged tissues, we assessed the dissemination of rTEM neutrophils following IR injury. For this purpose, we adapted our recently developed *in vivo* cell labeling method of tracking rTEM neutrophils, a technique that takes advantage of the strong affinity of biotin for streptavidin (Owen-Woods et al., 2020). Briefly, luminal neutrophils were stained via i.v. injection of a biotinylated anti-Ly6G mAb, followed by local application of AF647-streptavidin to inflamed tissues. Initial studies confirmed that while this strategy did not significantly label luminal neutrophils, it definitively labeled all neutrophils that had breached EC junctions during IR injury. As such, luminal neutrophils were streptavidin^{lo}, whereas interstitial and rTEM neutrophils were streptavidin^{hi} (Figures 5E; Figures S5A, S5B, and Video S4). With this technique, sham mice presented low levels of streptavidin^{hi} neutrophils in blood. In contrast, IR-treated mice exhibited significant dissemination of locally generated rTEM neutrophils in the peripheral circulation at 1 h post reperfusion (Figures 5F and 5G). In line with their greater frequency of rTEM neutrophils (Figure 5D), aged animals showed an elevated level of streptavidin^{hi} neutrophils in blood (corresponding to ~5,000 and ~50,000 cells/mL of blood in young and aged animals, respectively). This response returned to baseline following 4 h reperfusion in both young and aged mice (Figure 5G), suggesting trafficking of rTEM streptavidin^{hi} neutrophils to other organs, most likely the BM, as demonstrated previously (Owen-Woods et al., 2020). Although young mice exhibited no retention of streptavidin^{hi} neutrophils in lungs, aged mice showed a dramatic enrichment of rTEM neutrophils in their pulmonary vasculature at 1 h post reperfusion (Figures 5H and 5I). Also evident at 4 h post reperfusion, these results suggest a sustained accumulation or retention of streptavidin^{hi} rTEM neutrophils in the lungs of aged animals. Together, following acute local injury, aged tissues prompt a remarkable level of transmigrating neutrophils to re-enter the blood circulation, cells that disseminate to the lungs where they are retained.

rTEM neutrophils are programmed toward an activated state in aged lungs and are directly noxious to the lung tissue

To directly investigate the tissue damaging potential of rTEM cells, we analyzed the phenotype of streptavidin^{hi} rTEM neutrophils. Using blood samples collected at 1 h post reperfusion of cremasteric tissues in young and aged mice, streptavidin^{hi} neutrophils exhibited minor phenotypic changes in both age groups, compared to streptavidin^{lo} neutrophils (Figures S6A and S6B). These findings reflected the overall phenotype of streptavidin^{lo} blood neutrophils that was not significantly different between sham and IR-treated groups in both young and aged mice (Figure S6C). Since we detected increased retention of streptavidin^{hi} neutrophils in the pulmonary vasculature of aged mice (Figure 5I), we hypothesized that the pro-inflammatory state of the vasculature of aged lungs may contribute to the local tissue damaging capacity of rTEM neutrophils. To assess this, we analyzed the phenotype of pulmonary vascular streptavidin^{hi} versus streptavidin^{lo} neutrophils in aged animals. Here, we detected no significant phenotypic change in streptavidin^{hi} neutrophils at 1 h post reperfusion. In contrast, streptavidin^{hi} neutrophils of 4 h samples exhibited a marked activated state with significantly increased expression of CD11b, ICAM-1, neutrophil elastase, CD66a and CXCR4, and reduced expression of CD62L and CXCR2 (Figures 6A, 6B, and 6C). These results suggest that retention of rTEM neutrophils in the pulmonary vasculature of aged mice (Figures 5H and 5I) leads to progressive activation of these cells. We detected no change in phenotype of streptavidin^{lo} cells when comparing sham and IR groups of young and aged mice (Figure S6D). This indicates that the enhanced activation state of pulmonary neutrophils was restricted to the streptavidin^{hi} neutrophil population, findings that are similar to those detected in blood neutrophils (Figure S6C). Together, these data preclude the possibility that, in IR–treated mice, circulating soluble factors determine the phenotype of the streptavidin^{hi} neutrophils and show that these cells have no impact on the phenotype of streptavidin^{lo} neutrophils.

Having found that rTEM neutrophils are retained and programmed toward an activated state in aged lungs, we conducted adoptive cell transfer experiments to directly investigate the potential tissue damaging impact of this scenario (Figure 6D). We FACS sorted streptavidin^{lo} and streptavidin^{hi} neutrophils from blood of young or aged mice subjected to IR injury at 1 h post reperfusion, cells that showed similar phenotypes and little evidence of activation (Figures S6A and S6B). The sorted cells were injected i.v. into unstimulated aged animals, and 4 or 24 h later, the mice were analyzed for multiple organ damage. Irrespective of the donor age, aged recipients injected with streptavidin^{hi} neutrophils exhibited significantly elevated lung permeability responses at 4 h, as compared to mice injected with streptavidin^{lo} cells (Figures 6E and 6F). Assessment of lung permeability at 24 h post injection of streptavidin^{hi} neutrophils (sorted from aged donors) indicated a sustained injury level (~31% increase in permeability; Figure 6F), suggesting limited

(D) Percentage of luminal CXCR2^{lo} neutrophils (n = 3-4 mice/group).
(E) Total neutrophil TEM events and (F) frequency of neutrophil rTEM as assessed by confocal IVM (n = 3-4 mice/group). Means ± SEM #p < 0.05, ####p < 0.0001 as compared to young, *p < 0.05, ***p < 0.001 as indicated.
See also Figure S4.

CellPress
OPEN ACCESS

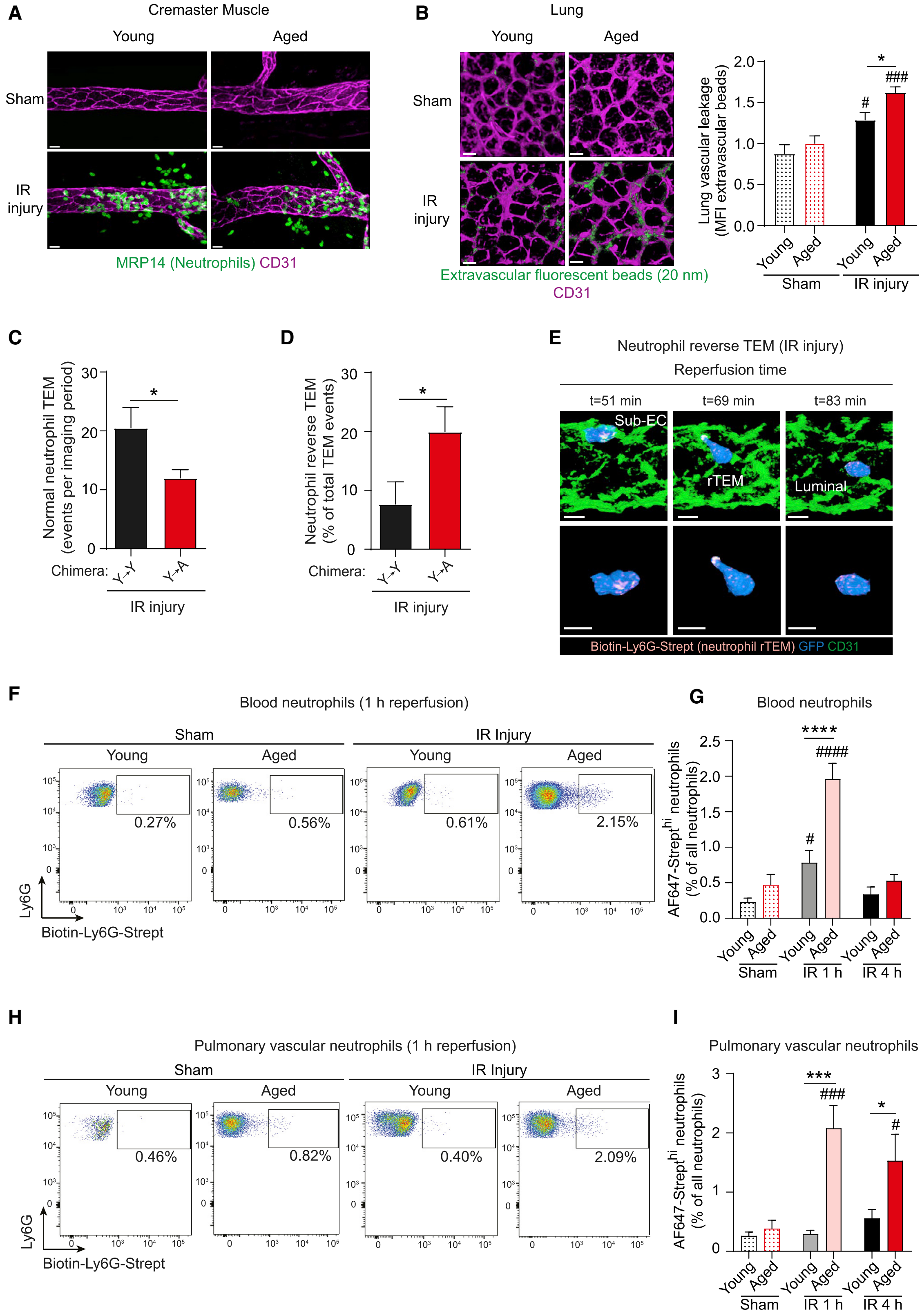

Figure 5. rTEM neutrophils stemming from locally injured aged tissues accumulate in the lungs.
Young and aged mice were subjected to sham or cremasteric IR injury.
(A) Representative confocal images of post-capillary venules (PCVs) immunostained for CD31 and MRP14 (neutrophils) in WT mice (scale bar: 20 μm).

(legend continued on next page)

recovery from the damage. Since streptavidin^{hi} neutrophils were retained in the pulmonary vasculature of aged mice (Figure 5I), we hypothesized that aged tissue is the determining factor in the injurious effect of rTEM neutrophils. To test this notion, we analyzed the effect of i.v. streptavidin^{hi} neutrophils (sorted from aged donors) in unstimulated young recipients. We saw no difference in lung permeability in mice injected i.v. with streptavidin^{hi} neutrophils as compared to mice injected with streptavidin^{lo} or PBS alone (Figure 6G), indicating the critical role of aged lung tissue in programming streptavidin^{hi} neutrophils toward a noxious phenotype. We detected no change between any of the cohorts of mice with respect to permeability in the liver, heart, gut, brain, or kidneys (Figure S7). Taken together, in aged animals, rTEM neutrophils constitute a population of cells that home to the lungs where they are programmed toward an activated state and are capable of directly inducing tissue damage.

CXCL1 blockade protects aged mice from remote organ damage

Finally, having identified increased CXCL1 in EC junctions as a driver of neutrophil rTEM in aged animals, and since rTEM neutrophils can directly cause lung damage in aged mice (Figure 6E), we considered that blockade of CXCL1 may be a strategy for protecting against aging-associated lung damage. This was investigated in the context of downstream pathological impact of local IR injury. Briefly, mice were treated i.v. with a blocking anti-CXCL1 or isotype control mAb at the time of reperfusion and were assessed for lung permeability. While following local IR aged mice exhibited an elevated lung vascular leakage response as compared to young, this increase was abrogated with the anti-CXCL1 mAb (Figure 7). These results suggest that pathways involved in driving neutrophil rTEM in aged conditions, such as dysregulated generation of directional cues, could be amenable to pharmacological blockade to protect aged individuals from developing acute lung damage post local injury.

DISCUSSION

Inflammation contributes to immune defenses, but when dysregulated, as is commonly observed in aging, it becomes a major detriment to normal physiological functions and health. Indeed, inflammation constitutes a major element of the aging process that is characterized by a low grade chronic inflammatory state and a shift toward increased cytokine and chemokine levels in tissues (López-Otín et al., 2013; Nikolich-Žugich, 2018). The immense functional implications of this highlight the need for greater mechanistic understanding of the impact of age on immunological processes. Here, we report on the occurrence of neutrophil reverse TEM in inflamed aged tissues, an aberrant event leading to downstream remote organ injury. Mechanistically, this response was caused by a heightened inflammatory vascular milieu as elicited by defined local cellular and molecular changes. Together, we show that elucidation of deleterious mechanisms within aged tissues can identify therapeutic targets aimed at normalizing local injurious neutrophil trafficking and preventing remote organ damage in the elderly population.

Hypothesizing that aging-associated inflammation perturbs immune cell dynamics with pathological consequences, here, we analyzed neutrophil trafficking *in vivo*, as investigated by high resolution confocal IVM. In aged mice, we observed a high frequency of neutrophil retrograde motility within EC junctions whereby neutrophils that had initiated diapedesis into inflamed tissues subsequently reverse migrated back to the venular lumen. Such neutrophil reverse TEM is seen *in vitro* using cultured ECs (Buckley et al., 2006), in zebra fish embryos (Mathias et al., 2006), and in certain locally inflamed mouse models (Colom et al., 2015; Owen-Woods et al., 2020; Woodfin et al., 2011). Neutrophil rTEM is aligned with downstream detrimental effects to remote organs, presenting this phenomenon as a cellular means of disseminating a local inflammatory response (Colom et al., 2015; Owen-Woods et al., 2020; Woodfin et al., 2011). As distant organ damage following local insults constitutes a significant medical problem in the elderly, the detection of increased neutrophil rTEM in aged tissues may represent a previously unknown element of aging-associated pathologies. This response was strictly driven by the aged tissue demonstrating that potential cell-intrinsic defects in neutrophil motility are unlikely to contribute to induction of rTEM. *In vitro* studies suggest defective chemotaxis of neutrophils isolated from aged individuals, an effect restored by inhibition of PI3K activity (Sapey et al., 2014). Given the vital role of PI3K in neutrophil interstitial migration (de Oliveira et al., 2016), even though potential flawed neutrophil chemotaxis does not appear to compromise neutrophil TEM, it may lead to inefficient directional interstitial motility in aged tissues. In this context, neutrophil interstitial migration toward the core of an inflammatory insult is mediated by neutrophil swarming, a response choreographed by sequential phases of highly coordinated cellular behaviors, most notably chemotaxis (Kienle and Lämmermann, 2016).

Mechanistically, neutrophil elastase cleavage of the EC junctional adhesion molecule, JAM-C, and disrupted localized presentation of chemotactic cues are triggers of neutrophil rTEM (Colom et al., 2015; Girbl et al., 2018; Owen-Woods et al., 2020; Woodfin et al., 2011). Here, we report that induction of neutrophil rTEM within aged venules was attributed to a heightened tissue inflammatory milieu, in particular, to increased levels of the ELR^{+} CXC chemokine, CXCL1. Among the most potent inducers of neutrophil trafficking, CXCL1 is released by stimulated vascular and

(B) Representative confocal images and quantification of lung vascular leakage in WT mice 4 h post reperfusion (scale bar: 20 μm; n = 4-5 mice/group).
(C) Neutrophil normal TEM events and (D) frequency of neutrophil rTEM in Y→Y or Y→A chimeras (see Figure 1H) as assessed by confocal IVM (n = 6 mice/group).
(E-I) Mice were injected i.v. with a biotinylated anti-Ly6G mAb and AF647-Strept locally applied to the cremaster muscle.
(E) Time-lapse confocal IVM images (Video S4) of a neutrophil rTEM event in an aged *Lyz2-EGFP-ki* cremaster muscle during IR injury illustrating that the neutrophil exhibiting rTEM is AF647-Strept^{hi} (Top panel: *en face* luminal view; bottom panel: isolated neutrophil; scale bar: 4 μm).
(F-I) Representative flow cytometry profiles and frequency of AF647-Strept^{hi} neutrophils in (F-G) blood and (H-I) pulmonary vascular washouts in WT mice (n = 4-11 mice/group). Numbers indicate the percentage of AF647-Strept^{hi} neutrophils. Means ± SEM, #p < 0.05, ###p < 0.001, ####p < 0.0001 as compared to age-matched controls, *p < 0.05, ***p < 0.001, ****p < 0.0001 as indicated.
See also Figure S5.

CellPress
OPEN ACCESS

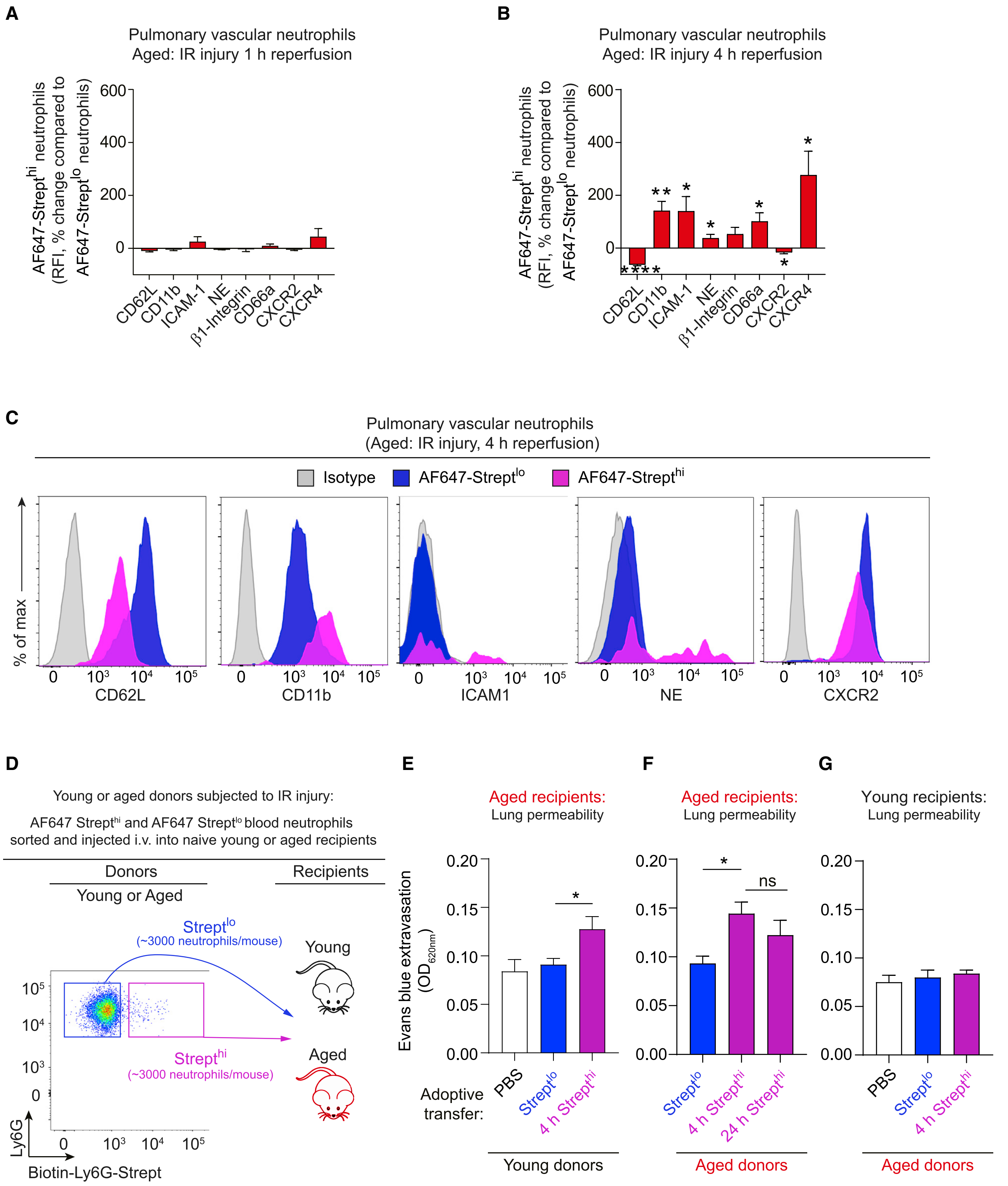

Figure 6. rTEM neutrophils are programmed toward an activated state in aged lungs and are directly noxious to the lung tissue

(A–C) Young and aged WT mice were injected i.v. with biotinylated anti-Ly6G mAb, subjected to sham or cremasteric IR injury and AF647-Strept applied locally to the cremaster muscle. Expression levels of indicated markers on AF647-Strept^{hi} neutrophils relative to levels on AF647-Strept^{lo} neutrophils within the pulmonary vasculature (A) 1 h or (B) 4 h post-reperfusion (n = 5–9 mice/group) and (C) representative histograms of indicated markers on pulmonary vascular neutrophils of aged mice 4 h post-reperfusion.

(D) Flow cytometry sorting strategy of AF647-Strept^{lo} and AF647-Strept^{hi} neutrophils from whole blood of young or aged mice 1 h post-reperfusion and subsequent i.v. injection into naive young or aged mice.

(legend continued on next page)

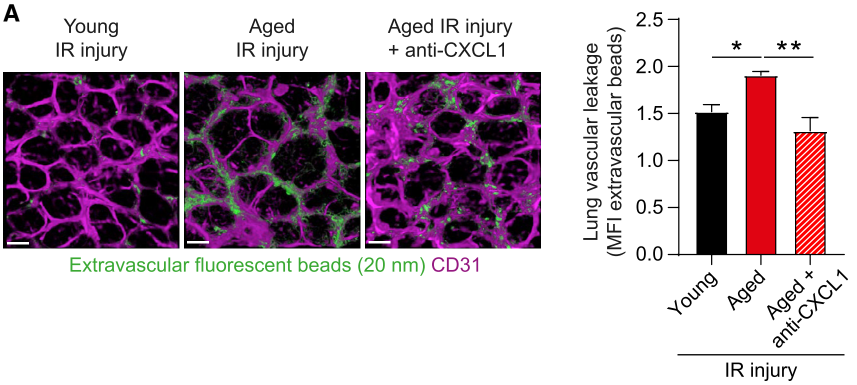

Figure 7. CXCL1 blockade protects aged mice from excessive lung injury

(A) Representative confocal images of whole mount lung (scale bar: 20 μm) and (B) lung vascular leakage quantification in young and aged WT mice subjected to sham or cremasteric IR injury and treated with an isotype control or anti-CXCL1 blocking mAb (n = 4 mice/group). Means ± SEM. *p < 0.05, **p < 0.01 as indicated.

perivascular cells. In this study we identified mast cells as a principal source of excessive CXCL1 and driver of neutrophil rTEM in inflamed aged tissues. The significant role of mast cells in this phenomenon appeared to be regulated at multiple levels. Aged tissues exhibited increased numbers of mast cells in close apposition to venular walls, a finding that is in agreement with data from aged human skin (Gunin et al., 2011; Pilkington et al., 2019). Mast cells of stimulated aged tissues were an abundant source of CXCL1, a cellular feature that was not evident in young tissues. Considering potential causes of this, mast cells of aged mice presented numerous elements of cellular senescence, suggesting that their enhanced capacity to generate CXCL1 may be linked to a cell type- and context-dependent SASP. The SASP represents one of a multitude of responses aligned with cellular senescence, a state of permanent cell-cycle arrest that can be induced by a range of exogenous and endogenous stresses (Franceschi and Campisi, 2014). While the properties and causes of mast cell senescence require further explorations, the oxidative stress environment of aged tissues is a key inducer of senescence (Franceschi and Campisi, 2014). The SASP may also facilitate enhanced vascular permeability through increased release of mast cell-derived pro-permeability mediators (e.g., histamine and LTC4), the former being a response that aligns with induction of neutrophil rTEM (Owen-Woods et al., 2020). The pro-inflammatory state and altered structural changes of aged tissues may additionally induce exaggerated responsiveness of tissue resident cells by providing co-stimulatory signals. An example is increased levels of extracellular matrix molecules (ECMs) (Kular et al., 2014; Labat-Robert, 2004) that could prime cells for elevated effector functions, and indeed, mast cells adherent to ECMs release greater levels of pro-inflammatory mediators. Moreover, ECMs may act as local cellular docking substrates (Krüger-Krasagakes et al., 1999; Oki et al., 2006), a mechanism that could contribute to increased retention and survival of mast cells and hence their increased number in aged organs.

Appropriate regulation of cellular dynamics within the complex 3-dimensional structures of the vascular system, lymphoid and non-lymphoid organs is a fundamental element of effective physiological immunity (Germain et al., 2012; Nourshargh and Alon, 2014; Weninger et al., 2014). In the context of neutrophil diapedesis, this is exquisitely regulated by correct spatial and temporal localization of chemokines within stimulated venular walls (Girbl et al., 2018; Nourshargh and Alon, 2014). Hypothesizing that excessive mast cell-derived CXCL1 disrupts the correct vascular localization of this chemokine, we noted enhanced presentation of CXCL1 at EC junctions of aged venules. This retention was entirely mediated by ACKR1 that was expressed at elevated levels in aged post-capillary venules. While ACKR1 contributes to the regulation of chemokine availability on ECs and leukocyte diapedesis (Girbl et al., 2018; Pruenster et al., 2009), here, we report that aging-associated increased protein expression of EC ACKR1 was detrimental to normal physiological neutrophil diapedesis. Specifically, EC ACKR1 played an indispensable role in retention of mast cell-derived CXCL1 at EC contacts. Additionally, it facilitated downregulation of CXCL1's cognate receptor, CXCR2, on transmigrating neutrophils. Together, these sequential events promoted neutrophil rTEM, thus identifying increased protein level of ACKR1 as a significant element of aberrant neutrophil trafficking in inflamed aged tissues. Hence, although downregulation of neutrophil GPCRs provides a physiological mechanism that fine tunes neutrophil migration and effector functions in response to surrounding stimuli, their sustained refractory state can result in dysregulated or aborted cell migration. This was illustrated *in vivo* through the use of chimeric mice expressing GRK2 deficient neutrophils. GRK2 is a G protein receptor kinase necessary for the phosphorylation and internalization of CXCR2 (Aragay et al., 1998; Raghuwanshi et al., 2012), and its deletion in neutrophils had no impact on TEM initiation indicating that pre-TEM adhesion events are not regulated by CXCR2 desensitization. However, neutrophil GRK2 deficiency led to complete abolition of neutrophil rTEM in aged mice, confirming CXCR2 desensitization as the causal trigger of this aberrant response. Together, our findings

(E–G) Extravasation of i.v. Evans blue in lung tissue in (E) aged recipients 4 h post i.v. injection of PBS or neutrophils sorted from young donors, (F) aged recipients 4 h or 24 h post i.v. injection of neutrophils sorted from aged donors, and (G) young recipients 4 h post i.v. injection of PBS or neutrophils sorted from aged donors (n = 4–7 mice/group). Means ± SEM. *p < 0.05, **p < 0.01, ****p < 0.0001, n.s. not significant as indicated or as compared to AF647-Strept^{lo} neutrophils of the same group.

See also Figures S6 and S7.

CellPress
OPEN ACCESS

demonstrate that ACKR1-mediated pro-inflammatory state of aged venules instigates excessive ligation, and hence desensitization, of neutrophil CXCR2, resulting in loss of neutrophil directional motility within EC junctions.

Collectively, we describe a cascade of events that elicit dysregulated neutrophil migration through aged venular walls. Essential components of this are changes in the local inflammatory milieu, namely, (i) an increased number and activation state of mast cells that underpin elevated tissue levels of CXCL1, and (ii) an increased protein level of EC ACKR1. As such, while the role of mast cells in innate and adaptive immunity is well acknowledged (Galli et al., 2020), our findings support the concept that these tissue-resident immune cells contribute to the pro-inflammatory state of aged tissues. Additionally, we have identified mast cells as key regulators of the dynamics of neutrophil trafficking in aging. Since mast cell numbers are increased in inflammatory disorders, such as asthma, rheumatoid arthritis and psoriasis (Siebenhaar et al., 2018), the mechanistic insights provided here may extend to chronic inflammatory conditions. With respect to ACKR1, while its expression on erythrocytes is considered as a sink or reservoir for inflammatory chemokines in the circulation and EC ACKR1 plays a crucial role in mediating leukocyte trafficking (Girbl et al., 2018; Novitzky-Basso and Rot, 2012), the mechanisms that regulate ACKR1 expression and function require further exploration. Here, since cytokine stimulation induced upregulation of EC ACKR1 protein in both young and aged tissues, EC ACKR1 expression appears to be transcriptionally regulated. Furthermore, this effect is likely to be exaggerated by the heightened pro-inflammatory state of aged tissues. The mechanisms that determine junctional localization of ACKR1 however remain unexplored. Similar to mast cell numbers, ACKR1 protein expression is increased in chronic inflammatory settings, as indicated by its upregulation on ECs in CNS microvessels during experimental and human multiple sclerosis (Minten et al., 2014). The molecular basis of the tissue aberrations reported here are likely very complex and representative of tissue-level adaptations to the pro-inflammatory stress state of aged stroma. Hence, we can speculate that altered mast cell numbers and phenotype may represent a compensation for aging-associated defective local immune and wound healing mechanisms. Similarly, increased expression of vascular ACKR1 may be a homeostatic adaptation to aging-associated exaggerated local and systemic inflammation.

Linking neutrophil rTEM to remote organ injury (Colom et al., 2015; Owen-Woods et al., 2020; Woodfin et al., 2011) indicates an important pathomechanistic role for this migratory response. Extending this paradigm, here we provide direct evidence for noxious capability of rTEM neutrophils and offer neutrophil rTEM as a novel mechanistic component of distant organ damage in aging. The local mechanisms linked to the induction of neutrophil rTEM are highly amenable to therapeutic targeting (e.g., involvement of CXCR2 ligands, mast cells, and GRK2), and as such, present new potential opportunities of suppressing remote organ damage in elderly individuals. Although details of how rTEM neutrophils induce tissue damage remain to be determined, rTEM neutrophils retained in lungs exhibited an adhesive phenotype and expressed elevated cell surface levels of neutrophil elastase and CXCR4. These molecular changes can collectively contribute to the trafficking, enrichment, and tissue destructive capability of rTEM neutrophils in lungs (Németh et al., 2020; Wang et al., 2017). As the homing and retention of rTEM neutrophils in aged lungs was instrumental in their programming toward an activated state, elucidating the molecular basis of this process will be of particular interest. The lung microvasculature is a significant depot for neutrophils, supporting neutrophil-mediated host defense and immunoregulation of activated, primed, and aged neutrophils (Granton et al., 2018). Aiming to delineate lung-specific recruitment mechanisms, dipeptidase-1 is a regulator of neutrophil homing to lungs (Choudhury et al., 2019), but whether the expression of this molecule is regulated by aging is unknown. Additionally, mouse single cell transcriptomic data (Consortium et al., 2018; Kalucka et al., 2020) hold immense promise in identifying organ-specific mechanisms of immune cell trafficking that can be subsequently explored in aged tissues.

In summary, despite the advances in neutrophil biology and the greater understanding of the neutrophil's role in immune pathophysiology, development of therapeutic strategies aimed at suppressing neutrophil-mediated tissue damage, without compromising immunity, has made little progress. However, targeting the generation and or function of noxious neutrophil "subsets" is emerging as a plausible means of controlling neutrophil-mediated disease on-set and progression (Beyrau et al., 2012; Ng et al., 2019; Scapini et al., 2016). Here, we present evidence for neutrophils that exhibited rTEM as one such specific neutrophil subpopulation with great tissue-destructive potential. The mechanistic insights delivered by this work suggest possible therapeutic avenues for suppression of aging-associated pathologies and provide a deeper understanding of immune cell trafficking in the broader context of chronic inflammatory disorders.

LIMITATIONS OF STUDY

Having identified altered number and phenotype of mast cells as key elements of increased frequency of neutrophil rTEM in aged tissues, determining the underlying mechanisms of these cellular events will be a critical goal. Such works could involve assessing the impact of age on the number of circulating mast cell precursors and local levels of mast cell growth factors. Furthermore, while the findings of this study have immense implications for aging-associated pathologies, at present there is no evidence for increased occurrence of neutrophil rTEM in aged individuals. To address this important limitation, defining the molecular signature of murine and human neutrophils at single cell level post exhibiting reverse TEM will be a key objective of future studies. Such works will act as a crucial prerequisite for the detection and functional evaluation of rTEM neutrophils in a broader range of physiological and pathological inflammatory settings in humans and experimental systems. Finally, establishing the molecular basis of rTEM neutrophil retention in lungs and the mechanism through which they are programmed toward an activated and tissue-damaging neutrophil sub-set by genetic and pharmacological means will be critical avenues to explore.

STAR★METHODS

Detailed methods are provided in the online version of this paper and include the following:

- KEY RESOURCES TABLE
- RESOURCE AVAILABILITY
  - Lead contact
  - Materials availability
  - Data and code availability
- EXPERIMENTAL MODELS AND SUBJECT DETAILS
  - Animal experimental models
- METHOD DETAILS
  - Generation of bone marrow chimeric animals
  - Inflammatory response in cremaster muscles
  - Inflammatory response in the ear skin
  - IF staining of whole mount tissues
  - Confocal microscopy and image analysis
  - Quantification of tissue chemokine content
  - Cremaster muscle intravital microscopy
  - Ear skin multiphoton intravital microscopy
  - Remote organ vascular leakage quantification
  - Flow cytometry
  - Tracking and phenotyping of rTEM neutrophils
  - Neutrophil GRK2 protein expression analysis
  - Mast cell depletion
  - Mast cell collection and analysis
- QUANTIFICATION AND STATISTICAL ANALYSIS

SUPPLEMENTAL INFORMATION

Supplemental information can be found online at https://doi.org/10.1016/j.immuni.2021.04.025.

ACKNOWLEDGMENTS

This work was funded by the Wellcome Trust (098291/Z/12/Z to S.N.). Additionally, the study was supported by funds from the British Heart Foundation (PG/17/85/33395 to S.N. R.J. and M.-B.V.), the People Programme (Marie Curie Actions) of the EU's 7th Framework Programme (FP7/2007-2013) under REA grant agreement (n° 608765) (to L.R., R.J., and S.N.) and the Deutsche Forschungsgemeinschaft SFB1123-A10 (to J.D.). We thank Dr T.J. Williams for critical assessment.

AUTHOR CONTRIBUTIONS

Conceptualization and writing (first draft): A.B., L.R. and S.N.; Methodology, investigation, analysis, and validation: S.N., A.B., L.R., R.J., J.B., C.O.-W., T.L., T.G., M-B.V., C.L.B., N.R-R., M.S., L.V.M., M.G.; Resources: R.N.P., R.S.S., A.T., U.H.v.A., J.D., C.L.B., D.V., A.R., A.R., T.L.; Editing of draft: All authors; Overall project conception, supervision, and funding: S.N.

DECLARATION OF INTERESTS

The authors declare no competing interests.

Received: October 9, 2020
Revised: March 11, 2021
Accepted: April 27, 2021
Published: May 24, 2021

REFERENCES

Akbar, A.N., and Gilroy, D.W. (2020). Aging immunity may exacerbate COVID-19. Science *369*, 256–257.

Aragay, A.M., Ruiz-Gómez, A., Penela, P., Sarnago, S., Elorza, A., Jiménez-Sainz, M.C., and Mayor, F., Jr. (1998). G protein-coupled receptor kinase 2 (GRK2): mechanisms of regulation and physiological functions. FEBS Lett. *430*, 37–40.

Asai, K., Kitaura, J., Kawakami, Y., Yamagata, N., Tsai, M., Carbone, D.P., Liu, F.T., Galli, S.J., and Kawakami, T. (2001). Regulation of mast cell survival by IgE. Immunity *14*, 791–800.

Beyrau, M., Bodkin, J.V., and Nourshargh, S. (2012). Neutrophil heterogeneity in health and disease: a revitalized avenue in inflammation and immunity. Open Biol. *2*, 120134.

Brandt, E.B., Strait, R.T., Hershko, D., Wang, Q., Muntel, E.E., Scribner, T.A., Zimmermann, N., Finkelman, F.D., and Rothenberg, M.E. (2003). Mast cells are required for experimental oral allergen-induced diarrhea. J. Clin. Invest. *112*, 1666–1677.

Buckley, C.D., Ross, E.A., McGettrick, H.M., Osborne, C.E., Haworth, O., Schmutz, C., Stone, P.C., Salmon, M., Matharu, N.M., Vohra, R.K., et al. (2006). Identification of a phenotypically and functionally distinct population of long-lived neutrophils in a model of reverse endothelial migration. J. Leukoc. Biol. *79*, 303–311.

Choudhury, S.R., Babes, L., Rahn, J.J., Ahn, B.Y., Goring, K.R., King, J.C., Lau, A., Petri, B., Hao, X., Chojnacki, A.K., et al. (2019). Dipeptidase-1 Is an Adhesion Receptor for Neutrophil Recruitment in Lungs and Liver. Cell *178*, 1205–1221.e17.

Colom, B., Bodkin, J.V., Beyrau, M., Woodfin, A., Ody, C., Rourke, C., Chavakis, T., Brohi, K., Imhof, B.A., and Nourshargh, S. (2015). Leukotriene B4-Neutrophil Elastase Axis Drives Neutrophil Reverse Transendothelial Cell Migration In Vivo. Immunity *42*, 1075–1086.

Consortium, T.M.; Tabula Muris Consortium; Overall coordination; Logistical coordination; Organ collection and processing; Library preparation and sequencing; Computational data analysis; Cell type annotation; Writing group; Supplemental text writing group; Principal investigators (2018). Single-cell transcriptomics of 20 mouse organs creates a Tabula Muris. Nature *562*, 367–372.

Dawson, T.C., Lentsch, A.B., Wang, Z., Cowhig, J.E., Rot, A., Maeda, N., and Peiper, S.C. (2000). Exaggerated response to endotoxin in mice lacking the Duffy antigen/receptor for chemokines (DARC). Blood *96*, 1681–1684.

De Filippo, K., Dudeck, A., Hasenberg, M., Nye, E., van Rooijen, N., Hartmann, K., Gunzer, M., Roers, A., and Hogg, N. (2013). Mast cell and macrophage chemokines CXCL1/CXCL2 control the early stage of neutrophil recruitment during tissue inflammation. Blood *121*, 4930–4937.

de Oliveira, S., Rosowski, E.E., and Huttenlocher, A. (2016). Neutrophil migration in infection and wound repair: going forward in reverse. Nat. Rev. Immunol. *16*, 378–391.

Duchene, J., Novitzky-Basso, I., Thiriot, A., Casanova-Acebes, M., Bianchini, M., Etheridge, S.L., Hub, E., Nitz, K., Artinger, K., Eller, K., et al. (2017). Atypical chemokine receptor 1 on nucleated erythroid cells regulates hematopoiesis. Nat. Immunol. *18*, 753–761.

Dudeck, A., Dudeck, J., Scholten, J., Petzold, A., Surianarayanan, S., Köhler, A., Peschke, K., Vöhringer, D., Waskow, C., Krieg, T., et al. (2011). Mast cells are key promoters of contact allergy that mediate the adjuvant effects of haptens. Immunity *34*, 973–984.

Eskan, M.A., Jotwani, R., Abe, T., Chmelar, J., Lim, J.H., Liang, S., Ciero, P.A., Krauss, J.L., Li, F., Rauner, M., et al. (2012). The leukocyte integrin antagonist Del-1 inhibits IL-17-mediated inflammatory bone loss. Nat. Immunol. *13*, 465–473.

Faust, N., Varas, F., Kelly, L.M., Heck, S., and Graf, T. (2000). Insertion of enhanced green fluorescent protein into the lysozyme gene creates mice with green fluorescent granulocytes and macrophages. Blood *96*, 719–726.

CellPress
OPEN ACCESS

Ferrucci, L., and Fabbri, E. (2018). Inflammageing: chronic inflammation in ageing, cardiovascular disease, and frailty. Nat. Rev. Cardiol. *15*, 505–522.

Franceschi, C., and Campisi, J. (2014). Chronic inflammation (inflammaging) and its potential contribution to age-associated diseases. J. Gerontol. A Biol. Sci. Med. Sci. *69* (*Suppl 1*), S4–S9.

Fulop, T., Larbi, A., Douziech, N., Fortin, C., Guérard, K.P., Lesur, O., Khalil, A., and Dupuis, G. (2004). Signal transduction and functional changes in neutrophils with aging. Aging Cell *3*, 217–226.

Galli, S.J., Gaudenzio, N., and Tsai, M. (2020). Mast Cells in Inflammation and Disease: Recent Progress and Ongoing Concerns. Annu. Rev. Immunol. *38*, 49–77.

Germain, R.N., Robey, E.A., and Cahalan, M.D. (2012). A decade of imaging cellular motility and interaction dynamics in the immune system. Science *336*, 1676–1681.

Girbl, T., Lenn, T., Perez, L., Rolas, L., Barkaway, A., Thiriot, A., Del Fresno, C., Lynam, E., Hub, E., Thelen, M., et al. (2018). Distinct Compartmentalization of the Chemokines CXCL1 and CXCL2 and the Atypical Receptor ACKR1 Determine Discrete Stages of Neutrophil Diapedesis. Immunity *49*, 1062–1076.e6.

Gomez, C.R., Hirano, S., Cutro, B.T., Birjandi, S., Baila, H., Nomellini, V., and Kovacs, E.J. (2007). Advanced age exacerbates the pulmonary inflammatory response after lipopolysaccharide exposure. Crit. Care Med. *35*, 246–251.

Granton, E., Kim, J.H., Podstawka, J., and Yipp, B.G. (2018). The Lung Microvasculature Is a Functional Immune Niche. Trends Immunol. *39*, 890–899.

Gunin, A.G., Kornilova, N.K., Vasilieva, O.V., and Petrov, V.V. (2011). Age-related changes in proliferation, the numbers of mast cells, eosinophils, and cd45-positive cells in human dermis. J. Gerontol. A Biol. Sci. Med. Sci. *66*, 385–392.

Kalucka, J., de Rooij, L.P.M.H., Goveia, J., Rohlenova, K., Dumas, S.J., Meta, E., Conchinha, N.V., Taverna, F., Teuwen, L.A., Veys, K., et al. (2020). Single-Cell Transcriptome Atlas of Murine Endothelial Cells. Cell *180*, 764–779.e20.

Kienle, K., and Lämmermann, T. (2016). Neutrophil swarming: an essential process of the neutrophil tissue response. Immunol. Rev. *273*, 76–93.

Krüger-Krasagakes, S., Grützkau, A., Krasagakis, K., Hoffmann, S., and Henz, B.M. (1999). Adhesion of human mast cells to extracellular matrix provides a co-stimulatory signal for cytokine production. Immunology *98*, 253–257.

Kular, J.K., Basu, S., and Sharma, R.I. (2014). The extracellular matrix: Structure, composition, age-related differences, tools for analysis and applications for tissue engineering. J. Tissue Eng. *5*, 2041731414557112.

Kulkarni, U., Zemans, R.L., Smith, C.A., Wood, S.C., Deng, J.C., and Goldstein, D.R. (2019). Excessive neutrophil levels in the lung underlie the age-associated increase in influenza mortality. Mucosal Immunol. *12*, 545–554.

Labat-Robert, J. (2004). Cell-matrix interactions in aging: role of receptors and matricryptins. Ageing Res. Rev. *3*, 233–247.

Lämmermann, T., and Kastenmüller, W. (2019). Concepts of GPCR-controlled navigation in the immune system. Immunol. Rev. *289*, 205–231.

López-Otín, C., Blasco, M.A., Partridge, L., Serrano, M., and Kroemer, G. (2013). The hallmarks of aging. Cell *153*, 1194–1217.

Lord, J.M., Midwinter, M.J., Chen, Y.F., Belli, A., Brohi, K., Kovacs, E.J., Koenderman, L., Kubes, P., and Lilford, R.J. (2014). The systemic immune response to trauma: an overview of pathophysiology and treatment. Lancet *384*, 1455–1465.

Mathias, J.R., Perrin, B.J., Liu, T.X., Kanki, J., Look, A.T., and Huttenlocher, A. (2006). Resolution of inflammation by retrograde chemotaxis of neutrophils in transgenic zebrafish. J. Leukoc. Biol. *80*, 1281–1288.

Minten, C., Alt, C., Gentner, M., Frei, E., Deutsch, U., Lyck, R., Schaeren-Wiemers, N., Rot, A., and Engelhardt, B. (2014). DARC shuttles inflammatory chemokines across the blood-brain barrier during autoimmune central nervous system inflammation. Brain *137*, 1454–1469.

Németh, T., Sperandio, M., and Mócsai, A. (2020). Neutrophils as emerging therapeutic targets. Nat. Rev. Drug Discov. *19*, 253–275.

Ng, L.G., Ostuni, R., and Hidalgo, A. (2019). Heterogeneity of neutrophils. Nat. Rev. Immunol. *19*, 255–265.

Nikolich-Žugich, J. (2018). The twilight of immunity: emerging concepts in aging of the immune system. Nat. Immunol. *19*, 10–19.

Nomellini, V., Faunce, D.E., Gomez, C.R., and Kovacs, E.J. (2008). An age-associated increase in pulmonary inflammation after burn injury is abrogated by CXCR2 inhibition. J. Leukoc. Biol. *83*, 1493–1501.

Nomellini, V., Brubaker, A.L., Mahbub, S., Palmer, J.L., Gomez, C.R., and Kovacs, E.J. (2012). Dysregulation of neutrophil CXCR2 and pulmonary endothelial icam-1 promotes age-related pulmonary inflammation. Aging Dis. *3*, 234–247.

Nourshargh, S., and Alon, R. (2014). Leukocyte migration into inflamed tissues. Immunity *41*, 694–707.

Novitzky-Basso, I., and Rot, A. (2012). Duffy antigen receptor for chemokines and its involvement in patterning and control of inflammatory chemokines. Front. Immunol. *3*, 266.

Oki, T., Kitaura, J., Eto, K., Lu, Y., Maeda-Yamamoto, M., Inagaki, N., Nagai, H., Yamanishi, Y., Nakajima, H., Kumagai, H., and Kitamura, T. (2006). Integrin alphaIIbbeta3 induces the adhesion and activation of mast cells through interaction with fibrinogen. J. Immunol. *176*, 52–60.

Owen-Woods, C., Joulia, R., Barkaway, A., Rolas, L., Ma, B., Nottebaum, A.F., Arkill, K.P., Stein, M., Girbl, T., Golding, M., et al. (2020). Local microvascular leakage promotes trafficking of activated neutrophils to remote organs. J. Clin. Invest. *130*, 2301–2318.

Pilkington, S.M., Barron, M.J., Watson, R.E.B., Griffiths, C.E.M., and Bulfone-Paus, S. (2019). Aged human skin accumulates mast cells with altered functionality that localize to macrophages and vasoactive intestinal peptide-positive nerve fibres. Br. J. Dermatol. *180*, 849–858.

Pruenster, M., Mudde, L., Bombosi, P., Dimitrova, S., Zsak, M., Middleton, J., Richmond, A., Graham, G.J., Segerer, S., Nibbs, R.J., and Rot, A. (2009). The Duffy antigen receptor for chemokines transports chemokines and supports their promigratory activity. Nat. Immunol. *10*, 101–108.

Raghuwanshi, S.K., Su, Y., Singh, V., Haynes, K., Richmond, A., and Richardson, R.M. (2012). The chemokine receptors CXCR1 and CXCR2 couple to distinct G protein-coupled receptor kinases to mediate and regulate leukocyte functions. J. Immunol. *189*, 2824–2832.

Robba, C., Battaglini, D., Pelosi, P., and Rocco, P.R.M. (2020). Multiple organ dysfunction in SARS-CoV-2: MODS-CoV-2. Expert Rev. Respir. Med. *14*, 865–868.

Sapey, E., Greenwood, H., Walton, G., Mann, E., Love, A., Aaronson, N., Insall, R.H., Stockley, R.A., and Lord, J.M. (2014). Phosphoinositide 3-kinase inhibition restores neutrophil accuracy in the elderly: toward targeted treatments for immunosenescence. Blood *123*, 239–248.

Scapini, P., Marini, O., Tecchio, C., and Cassatella, M.A. (2016). Human neutrophils in the saga of cellular heterogeneity: insights and open questions. Immunol. Rev. *273*, 48–60.

Shaw, A.C., Goldstein, D.R., and Montgomery, R.R. (2013). Age-dependent dysregulation of innate immunity. Nat. Rev. Immunol. *13*, 875–887.

Siebenhaar, F., Redegeld, F.A., Bischoff, S.C., Gibbs, B.F., and Maurer, M. (2018). Mast Cells as Drivers of Disease and Therapeutic Targets. Trends Immunol. *39*, 151–162.

Thiriot, A., Perdomo, C., Cheng, G., Novitzky-Basso, I., McArdle, S., Kishimoto, J.K., Barreiro, O., Mazo, I., Triboulet, R., Ley, K., et al. (2017). Differential DARC/ACKR1 expression distinguishes venular from non-venular endothelial cells in murine tissues. BMC Biol. *15*, 45.

Vabret, N., Britton, G.J., Gruber, C., Hegde, S., Kim, J., Kuksin, M., Levantovsky, R., Malle, L., Moreira, A., Park, M.D., et al.; Sinai Immunology Review Project (2020). Immunology of COVID-19: Current State of the Science. Immunity *52*, 910–941.

Voehringer, D., Liang, H.E., and Locksley, R.M. (2008). Homeostasis and effector function of lymphopenia-induced "memory-like" T cells in constitutively T cell-depleted mice. J. Immunol. *180*, 4742–4753.

Wang, J., Hossain, M., Thanabalasuriar, A., Gunzer, M., Meininger, C., and Kubes, P. (2017). Visualizing the function and fate of neutrophils in sterile injury and repair. Science *358*, 111–116.

Weninger, W., Biro, M., and Jain, R. (2014). Leukocyte migration in the interstitial space of non-lymphoid organs. Nat. Rev. Immunol. *14*, 232–246.

Woodfin, A., Voisin, M.B., Beyrau, M., Colom, B., Caille, D., Diapouli, F.M., Nash, G.B., Chavakis, T., Albelda, S.M., Rainger, G.E., et al. (2011). The junctional adhesion molecule JAM-C regulates polarized transendothelial migration of neutrophils in vivo. Nat. Immunol. *12*, 761–769.

Wulfert, F.M., van Meurs, M., Kurniati, N.F., Jongman, R.M., Houwertjes, M.C., Heeringa, P., Struys, M.M., Zijlstra, J.G., and Molema, G. (2012). Age-dependent role of microvascular endothelial and polymorphonuclear cells in lipopolysaccharide-induced acute kidney injury. Anesthesiology *117*, 126–136.

STAR★METHODS

KEY RESOURCES TABLE

REAGENT or RESOURCE	SOURCE	IDENTIFIER
Antibodies		
Anti-mouse ACKR1 (clone 6B7)	(Thiriot et al., 2017)	N/A
Anti-mouse CD117 (c-Kit) Alexa Fluor 647 (Clone 2B8)	Biolegend	Cat# 105818; RRID: AB_493474
Anti-mouse CD117 (c-Kit) APC (Clone 2B8)	Biolegend	Cat# 123121; RRID: AB_313220
Anti-mouse CD16/32 Purified Antibody	Biolegend	Cat# 101301; RRID: AB_312800
Anti-mouse CD182 (CXCR2) PE (Clone SA044G4)	Biolegend	Cat# 149303; RRID: AB_2565691
Anti-mouse CD184 (CXCR4) Monoclonal Antibody (2B11), PerCP-eFluor 710	Thermo Fischer Scientific	Cat# 46-9991-80; RRID: AB_10670489
Anti-mouse CD31 (clone 390)	Thermo Fischer Scientific	Cat# 16-0311-85; RRID: AB_468933
Anti-mouse CD45 Pacific Blue (clone 30-F11)	Biolegend	Cat# 103126; RRID: AB_493535
Anti-mouse CD54 PE/Cy7 (Clone YN1/1.7.4)	Biolegend	Cat# 116121; RRID: AB_2715949
Anti-mouse CD62L Brilliant Violet 605 (Clone MEL-14)	Biolegend	Cat# 104437; RRID: AB_11125577
Anti-mouse c-Kit (CD117) (clone D13A2)	Cell Signaling Technology	Cat# 3074T; RRID: AB_1147633
Anti-mouse CXCL1 (polyclonal)	R&D systems	Cat# AF-453-NA; RRID: AB_354495
Anti-mouse CXCR2 Alexa Fluor 647 (clone SA044G4)	Biolegend	Cat# 149305; RRID: AB_2565693
Anti-mouse F4/80 Alexa Fluor 647 (Clone BM8)	Biolegend	Cat# 123121; RRID: AB_893492
Anti-mouse FcεRIα Pacific Blue (Clone MAR1)	Biolegend	Cat# 134313; RRID: AB_10612933
Anti-mouse GRK2	Genetex	Cat# GTX101682
Anti-mouse Ly6G Pacific Blue™ (Clone 1A8)	Biolegend	Cat# 127611 RRID: AB_1877212
Anti-mouse Ly6G Alexa Fluor 488 (Clone 1A8)	Biolegend	Cat# 127625; RRID: AB_1186108
Anti-mouse Ly6G Alexa Fluor 647 (clone 1A8)	Biolegend	Cat# 127610; RRID: AB_1134159
Anti-mouse Ly6G Biotin (Clone 1A8)	Biolegend	Cat# 127604; RRID: AB_2561339
Anti-mouse MRP14 (clone 2B10)	Gift from Dr N.Hogg (Francis Crick Institute, UK)	N/A
Anti-mouse/human CD11b Brilliant Violet 711 (Clone M1/70)	Biolegend	Cat# 101241; RRID: AB_11218791
Anti-mouse/rat CD29 PE/Cy7 (Clone HMβ1-1)	Biolegend	Cat# 102221; RRID: AB_528789
Anti-mouse CD206 Alexa Fluor 647 (clone C068C2)	Biolegend	Cat# 141711 ; AB_10900240
Anti-Neutrophil Elastase antibody	Abcam	Cat# ab68672; RRID: AB_1658868
Armenian Hamster IgG Isotype Ctrl PE/Cy7	Biolegend	Cat# 400921
Blocking anti-mouse CXCL1 (clone 48415)	R&D systems	Cat# MAB453; RRID: AB_2087696
Blocking anti-mouse CXCL2 (clone 40605)	R&D systems	Cat# MAB452; RRID: AB_2230058
Blocking anti-mouse IgG2a	Biolegend	NA
Depletion Anti-mouse CD117 (c-kit) Antibody Ultra-LEAF™ Purified (Clone ACK2)	Biolegend	Cat# 135131; RRID: AB_2571992
Depletion Anti-mouse IgG2b κ Isotype control (Clone ACK2)	Biolegend	Cat# 135131; RRID 2571992
Polyclonal goat anti-rabbit immunoglobulins/ HRP conjugated antibody	Agilent/Dako	Cat# P044801-2
Rat IgG2a, κ Isotype Ctrl PE (Clone RTK2758)	Biolegend	Cat# 400507
Rat IgG2b kappa Isotype Control (eB149/10H5), PerCP-eFluor 710	Thermo Fischer Scientific	Cat# 46-4031-80; RRID: AB_1834457
Rat IgG2b, κ Isotype Ctrl Antibody Brilliant Violet 711 (Clone RTK4530)	Biolegend	Cat#400653

(*Continued on next page*)

Continued

REAGENT or RESOURCE	SOURCE	IDENTIFIER
Biological Samples		
Bone marrow: mouse *Mrp8-Cre;Grk2*$^{f/f}$*;Lyz2-EGFP-ki*	Dr Tim Lämmermann (Max Planck Institute of Immunobiology and Epigenetics, Germany)	N/A
Critical commercial assay		
Alexa Fluor 488 antibody labeling kit	Thermo Fisher Scientific	Cat# A20181
Alexa Fluor 555 antibody labeling kit	Thermo Fisher Scientific	Cat# A20187
Alexa Fluor 647 antibody labeling kit	Thermo Fisher Scientific	Cat# A20186
Anti-Ly-6G MicroBeads UltraPure, mouse	Milteny biotech	Cat# 130-120-337
DyLight 405 antibody labeling kit	Thermo Fisher Scientific	Cat# 53021
Mouse CXCL1/KC DuoSet ELISA	R&D Systems	Cat# 453-05
Proteome Profiler Mouse Cytokine Array Kit, Panel A	R&D Systems	Cat# ARY006
Chemicals, Peptides, and Recombinant Proteins		
AF647-streptavidin	Thermo Fisher Scientific	Cat# S21374
Avidin, Alexa Fluor 488 conjugate	Thermo Fischer Scientific	Cat# A21370
Avidin, Egg White	Thermo Fisher Scientific	Cat# A2667
Bafilomycin	AlfaAesar	Cat# J67193
BSA, low endotoxin	Sigma-Aldrich	Cat# A9543
C12FDG (5-Dodecanoylaminofluorescein Di-β-D-Galactopyranoside)	Thermo Fischer Scientific	Cat# D2893
DAPI (4',6-Diamidino-2-Phenylindole, Dilactate)	Biolegend	Cat# 422801
Evans Blue	Sigma	Cat# E2129
FITC Annexin V	BD	Cat# 560931
Halt Protease and Phosphatase Inhibitor Cocktail (100X)	Thermo Fisher Scientific	Cat# 78440
Propidium iodide solution	Biolegend	Cat# 421301
Recombinant murine IL-1β	R&D Systems	Cat# 401-ML-005/CF
Recombinant murine TNF-α aa 80-235	R&D Systems	Cat# 410-MT-010/CF
Triton X-100	Sigma	Cat# T8787-100ML
Experimental Models: Organisms/Strains		
Mouse, *Ackr1*$^{-/-}$	(Dawson et al., 2000)	N/A
Mouse C57BL/6	Charles River laboratories	JAX 000664
Mouse, C57BL/6JRj	Janvier laboratories	Cat# SC-C57J-M
Mouse, *Lyz2-EGFP-ki*	Gift from Dr M. Sperandio (Ludwig Maximilians University Munich, Germany) (Faust et al., 2000)	N/A
Mouse, *Mcpt5-Cre-R-DTA*	Provided by Prof. Axel Roers (Medical Faculty Carl Gustav Carus, Technische Universität Dresden)	N/A
Software and Algorithms		
FlowJo v10	Tree Star	https://www.flowjo.com/
ImageJ	Wayne Rasband (NIH)	https://imagej.nih.gov/ij/

(*Continued on next page*)

Continued

REAGENT or RESOURCE	SOURCE	IDENTIFIER
Imaris v9	Bitplane	https://imaris.oxinst.com/packages
Prism v8	Graphpad	https://www.graphpad.com/scientific-software/prism/
Other		
123 eBeads™ counting beads	Thermo Fisher Scientific	Cat# 01-1234-42
FluoSpheres™; Carboxylate-Modified Microspheres, 0.02 μm, red fluorescent (580/605)	Invitrogen	Cat# F8786
Latex-Free Orthodontic Elastic Bands	Dental Aesthetics	Cat# UNL735-F
UltraComp eBeads™ Compensation Beads	ThermoFisher Scientific	Cat# 01-2222-42
Zombie Yellow™ Fixable Viability Kit	Biolegend	Cat# 423103

RESOURCE AVAILABILITY

Lead contact

Further information and requests for resources and reagents should be directed and will be fulfilled by the lead contact Sussan Nourshargh (s.nourshargh@qmul.ac.uk).

Materials availability

The supply of the following reagents and mice are subject to MTA agreements with the academics indicated in parentheses: Anti-ACKR1 mAb (Dr Ulrich H von Andrian); *Lyz2-EGFP-ki* mice (Dr Thomas Graf); *Mcpt5-Cre-R-DTA* (Dr Axel Roers).

Data and code availability

This study did not generate or analyze large datasets or codes.

EXPERIMENTAL MODELS AND SUBJECT DETAILS

Animal experimental models

Mice were used at ages described in the text (‘young’: 2-4 months, and ‘aged’: $\geq$16 months). Wild type (WT) C57BL/6J and C57BL/6JRj mice were purchased from Charles River laboratories (Margate, UK) and Janvier (Le Genest-Saint-Isle, France), respectively. For each experiment, WT strain-, aged and sex-matched mice were used. *Lyz2-EGFP-ki* mice were used with permission of Dr Thomas Graf (Center for Genomic Regulation and ICREA, Spain) and provided by Dr Markus Sperandio (LMU, Munich, Germany). These mice have a EGFP cDNA cassette knocked into the lysozyme M (Lyz2) locus to generate GFP^{+} myeloid cells (GFP^{bright} neutrophils, GFP^{dim} monocytes and macrophages) and were backcrossed with C57BL/6 mice for at least 8 generations (Faust et al., 2000). *Ackr1*$^{-/-}$ mice were backcrossed onto a C57BL/6 background for at least 11 generations (Dawson et al., 2000). *Mrp8-Cre;Grk2*$^{f/f}$*;Lyz2-EGFP-ki* and littermate control *Grk2*$^{fl/fl}$*;Lyz2-EGFP-ki* mice were bred at the Max Planck Institute of Immunobiology and Epigenetics, Freiburg, Germany. *Mcpt5-Cre-R-DTA* mice were generated in the Roers laboratory as previously described (Dudeck et al., 2011). Briefly, the *Cre* recombinase is expressed under the control of the mast cell protease (Mcpt) 5 promoter. These mice were bred with the R-DTA line (Voehringer et al., 2008), which expresses the diphtheria toxin selectively in these cells leading to the depletion of connective type tissue mast cells. All animals were group housed in individually ventilated cages under specific pathogen-free (SPF) conditions and a 12 h (h) light-dark cycle. Animals were humanely sacrificed via cervical dislocation at the end of experiments in accordance with UK Home Office regulations. Male mice were used for all studies, with the exception of some female mice used for senescence, apoptosis, and ear IVM experiments. All *in vivo* experiments were conducted at the William Harvey Research Institute, Queen Mary University of London, UK under the UK legislation for animal experimentation and in agreement with the UK Home Office Animals Scientific Procedures Act 1986 (ASPA).

METHOD DETAILS

Generation of bone marrow chimeric animals

To generate chimeras, recipient mice were lethally irradiated with two doses of 5 Gray (Gy), 4 h apart using a RadSource-2000 irradiator. Freshly isolated bone marrow from the femurs of young or aged *Lyz2-EGFP-ki* mice were transplanted into aged or young WT recipients. In similar experiments, bone marrow from WT mice was transplanted into *Ackr1*$^{-/-}$ or WT recipients to establish the requirement of non-hematopoietic expression of ACKR1. Mice exhibiting GRK2 deficiency in neutrophils and respective controls were generated by transferring bone marrow from *Mrp8-Cre;Grk2*$^{fl/fl}$*;Lyz2-EGFP-ki* or *Grk2*$^{fl/fl}$*;Lyz2-EGFP-ki* littermate controls

into WT recipients. The following day, 1.5 × 10^6 to 2 × 10^6 donor bone marrow cells were injected i.v. into each irradiated recipient. Engraftment efficiency was assessed 4-8 weeks post irradiation by flow cytometry as described below. Generally, all mice receiving *Lyz2-EGFP-ki* bone marrow displayed ≥95% GFP^{bright} neutrophils with similar neutrophil counts in peripheral blood. Bone marrow reconstitution of *Ackr1*$^{-/-}$ recipients was additionally determined by assessing ACKR1 expression on erythrocytes by flow cytometry. Chimeric mice were used 4–10 weeks post transplantation. Analysis of numerous relevant inflammatory, cellular, and molecular parameters revealed comparable results in inflamed cremaster muscles of irradiated chimeras as compared to control non-irradiated mice (non-chimeric mice) (Table S1). Furthermore, non-irradiated and irradiated (chimeric) aged mice exhibited similar numbers of mast cells in their ear skin (7,023 ± 1,001 and 7,430 ± 1,555 cells per mm^3 of tissue ± SEM, respectively, n = 4 mice/group).

Inflammatory response in cremaster muscles

Mice were briefly anaesthetized with 3% isoflurane and injected intrascrotally (i.s.) with IL-1β (50 ng), TNF (300 ng) or phosphate buffered saline (PBS) as vehicle control with or without fluorescent dye-conjugated anti-CD31 mAb (4 μg, clone 390, Thermo Fischer Scientific) to label endothelial cell (EC) junctions, in a 400 μl bolus for 2-4 h stimulation periods. For some intravital microscopy (IVM) experiments, cremasteric ischemia-reperfusion (IR) injury was induced as previously described (Colom et al., 2015; Woodfin et al., 2011), with ischemia induced in the exteriorized cremaster muscle of anaesthetized mice by the placement of two non-crushing metal clamps (Interfocus, Schwartz Micro Serrefine) at the base of the exteriorized tissue for 40 min (mins). Subsequently, the clamps were removed and tissue reperfusion allowed. Control sham operated mice underwent surgical procedures without induction of IR. For other experiments, ischemia was induced in a non-surgical manner by the application of two orthodontic bands around the intact testes and scrotum to occlude the vasculature for 40 min, followed by 1-24 h of reperfusion.

Inflammatory response in the ear skin

Mice were anaesthetized by intramuscular injection (i.m.) of an anesthetic mix containing ketamine and xylazine in PBS and ears were injected intradermally (i.d.) with IL-1β (50 ng), or PBS as vehicle control, together with fluorescently labeled anti-CD31 mAb (4 μg, clone 390, Thermo Fischer Scientific) to label EC junctions, within a 40 μl bolus injection. Tissues were commonly analyzed 2-4 h later as detailed below.

IF staining of whole mount tissues

Cremaster muscles and ears were dissected and fixed in 4% paraformaldehyde (PFA, Sigma-Aldrich) for 15-60 min at 4°C. Tissues were blocked for non-specific staining and permeabilized in PBS containing 25% fetal calf serum (FCS; Thermo Fischer Scientific) and 0.5% Triton X-100 (Sigma-Aldrich, 0.05% for CXCR2 staining) for 4-5 h at room temperature under gentle rotation.

Tissues were then incubated with unconjugated or fluorescent dye-conjugated primary antibodies in PBS containing 10% FCS overnight at 4°C. Subsequently, tissues were incubated with species specific fluorescent dye-conjugated secondary antibodies for 3 h at room temperature in PBS containing 10% FCS. Antibody conjugation to Alexa Fluor-488, −555, −647 or -Dylight 405 fluorophores was achieved using labeling kits according to manufacturers' instructions. Tissue samples were whole-mounted in PBS onto glass slides and imaged by confocal microscopy.

Confocal microscopy and image analysis

Immunostained post-capillary venules (diameter: 20-40 μm) of the cremaster muscle or ear skin were imaged using an upright Leica TCS SP5, Leica SP8 or an inverted Zeiss LSM 800 laser-scanning confocal microscope equipped with argon and helium lasers (488, 561 and 633 nm excitation wavelengths), a tunable white light laser or solid-state laser diodes (405, 488, 561 and 640 nm excitation wavelengths), respectively. Serial Z stacks of post-capillary venules were acquired using a dry 10x/0.3 objective lens, water dipping 20x/1.0 objective lens, oil immersion 40x/1.3 or 63x/1.4 objectives lenses. Resulting images of half vessels optically sectioned in the longitudinal orientation were reconstructed and analyzed by IMARIS software™ (Bitplane, Zurich, Switzerland). To capture fields up to 3 mm x 3 mm, tile scan acquisition was performed where necessary using 10% tile overlap; tiles were stitched and fused using the ZEN software (Zeiss, Germany). Venular ECs were identified by CD31, mast cells (MC) by avidin and/or CD117 and macrophages by CD206 and CD115. For the quantification of blood vessel density, serial Z stacks of 8 random areas of cremaster muscles was performed using a 10x/0.3 dry objective. Using Imaris, the total tissue volume was quantified, then, using the CD31 channel and the Imaris Surface function, blood vessels were reconstructed in 3D and blood vessel volumes quantified. Vascular density was finally calculated by dividing the blood vessel volume with the whole tissue volume. Expressions of endogenous CXCL1, CD31 and ACKR1 were determined using a polyclonal anti-CXCL1 antibody (Girbl et al., 2018), anti-CD31 and an anti-ACKR1 mAb (Thiriot et al., 2017), respectively. Quantification and localization of these molecules was determined by their mean fluorescence intensities (MFI) within (junctional) or outside of (non-junctional) an IMARIS generated isosurface of CD31. CXCL1 expression by MCs or macrophages was similarly determined using an isosurface generated on avidin or CD206 immunostaining, respectively. MC cellular and nuclear volumes were quantified within CD117 and DAPI isosurfaces generated on IMARIS, respectively. The number of perivascular MCs and macrophages was defined as number of cells within a 50 μm perimeter of a post-capillary venule. Membrane CXCR2 expression was visually determined by immunostaining with an anti-CXCR2 mAb, and in mice not exhibiting *Lyz2-EGFP-ki* neutrophils, neutrophils were identified using an anti-MRP-14 Ab. $CXCR2^{lo}$ neutrophils were defined as neutrophils with a MFI 25% less than the average MFI of luminal neutrophils within the same image. For each molecule of interest, immunoreactive protein expression was quantified from 4-12 images/tissue and expressed as MFI values minus the low background signal of tissues stained with specific isotype control antibodies.

Quantification of tissue chemokine content

Cremaster muscles were homogenized in PBS containing 0.1% Triton and 1% Halt Protease and Phosphatase Inhibitor cocktail and mechanically dissociated using the Precellys24 beat-beading system (Bertin Technologies, France). The cytokine and chemokine expression profiles of these samples (pooled from 3 mice/group) were analyzed using a Mouse Cytokine Array Panel A kit (R&D Systems, Abingdon, Oxford) as per manufacturers' instructions. The CXCL1 content of these tissues was further validated by ELISA (R&D Systems; sensitivity: 2 pg/mL). Chemokine content was expressed as per unit weight of tissue.

Cremaster muscle intravital microscopy

Neutrophil-vessel wall interactions were analyzed in the mouse cremaster muscle microcirculation by brightfield and confocal intravital microscopy (IVM). For brightfield IVM, mice were injected i.s. with IL-1β (50 ng), TNF (300 ng) or PBS vehicle control for 4 h, after which the animals were terminally anaesthetized followed by surgical exteriorization of the cremaster muscle. Post exteriorization, the tissues were pinned flat onto a viewing platform of a custom made heated stage (maintaining the mouse body temperature at 37°C) and the exteriorized muscle was kept warm and moist during surgery and the imaging period through continuous superfusion of warm Tyrode's salt solution (9.6 g/L Tyrodes salt and 12 mM $NaHCO_3$). Post-capillary venules of 20-40 μm in diameter were observed in real time using a 63x water dipping objective on a transmitted light upright fixed stage microscope (Axioskop FS, Carl Zeiss) with a digital CMOS camera (Hamamatsu). Quantification of leukocyte rolling and firm adhesion (luminal neutrophils stationary for ≥30 s) responses were analyzed within multiple vessel segments (3-5) of several vessels (3-5) per mouse.

To assess the mode and dynamics of neutrophil migration across blood vessel walls, confocal IVM was applied to the neutrophil reporter *Lyz2-EGFP-ki* mice (Girbl et al., 2018; Woodfin et al., 2011). For some experiments, chimeric mice generated through transfer of *Lyz2-EGFP-ki* bone marrow to recipient animals were used. In other experiments, C57BL6/JRj mice were injected i.v. with 1.5 × 10^7 bone marrow cells from young *Lyz2-EGFP-ki* donors immediately before exteriorization of the cremaster muscle. For these studies, mice were anaesthetized using 3% isoflurane, and cremaster muscles stimulated via i.s. injection of IL-1β (50 ng), or were treated with PBS vehicle control. Concomitantly, the mice were injected i.s. with Alexa Fluor 555-anti-CD31 mAb (4 μg, clone 390, Thermo Fischer Scientific) to label EC junctions, all for 2 h test periods. Alternatively, mice were subjected to cremasteric IR injury as described above and as previously detailed (Woodfin et al., 2011). Mice were then terminally anaesthetized by intraperitoneal (i.p.) administration of ketamine (100 mg/kg) and xylazine (10 mg/kg), and anesthesia maintained by i.m. injections of the same compounds. For IR experiments, the blood flow of the exteriorized muscle was occluded for 40 min by the placement of two non-crushing clamps to the base of the tissue 2 h after i.s. Alexa Fluor 555 anti-CD31 mAb administration. In some experiments, blocking antibodies to CXCL1, CXCL2 or isotype control mAbs (1 mg/kg; i.v.) were injected via a tail vein cannula 5 min into the image acquisition period. In some experiments, mice were subjected to local MC depletion protocols prior to imaging as described below. To label and track rTEM neutrophils during cremasteric IR injury, biotinylated-anti-Ly6G mAb (2 μg; i.v.) was injected 10 min prior to the induction of ischemia. At the onset of reperfusion, the cremaster muscle was superfused with Alexa Fluor-647-streptavidin (1 μg/mL in Tyrode's solution) for the entirety of the imaging period. Post-capillary venules with diameters of 20-40 μm were imaged for 1-2 h using an upright Leica SP5 or SP8 confocal laser scanning microscope, both equipped with a 20x/1.0 water-dipping objective lens. Using the 8,000 Hz (Hz) resonant Z-scanner, Z stacks of 0.7 μm optical sections were acquired at 1 min intervals. Typically, images were acquired as 300 × 130 × 35 μm segments, resulting in a voxel size of approximately 0.29 (x) x 0.29 (y) x 0.69 (z) μm. Image series were then assembled into videos using IMARIS software to show longitudinally sectioned 'half' vessels for clarity and to enable direct visualization of luminal neutrophil-endothelial cell interactions. The mode and dynamics of neutrophil migration was determined by manual tracking of individual neutrophils using IMARIS. Normal neutrophil TEM (nTEM) was defined as a TEM event during which neutrophils fully breached EC junctions in a luminal-to-abluminal manner without pause. Neutrophil reverse TEM (rTEM) was classified as a response whereby neutrophils engaged with EC junctions, and after partial or full TEM, the cell retracted, exhibited retrograde motility and ultimately re-entered the vascular lumen. Neutrophil normal and reverse TEM events were quantified over an observation period of 60-120 min with the latter being expressed as a percentage of total TEM events observed during the same period. Neutrophil extravasation into the interstitium per field of view was quantified by manual counting at the end of the IVM imaging period.

Ear skin multiphoton intravital microscopy

Neutrophil-vessel wall interactions were analyzed in the mouse ear skin microcirculation by multiphoton intravital microscopy (IVM), as supported by the CMR Advanced Bio-imaging Facility at QMUL. Mice were briefly anaesthetized by i.m. injection of anesthetic mix (ketamine and xylazine in PBS) and ears were injected with i.d. IL-1β (50 ng) and Alexa Fluor 488-anti-CD31 mAb (4 μg, clone 390, Thermo Fischer Scientific) in a 40 μl bolus for a 2 h stimulation period. Thirty minutes before imaging, mice were injected i.v. with Alexa Fluor 647 anti-Ly6G mAb (1.5 μg, clone 1A8) to label luminal neutrophils. Mice were terminally anaesthetized and the ear skin pinned out flat onto a viewing platform of a custom made heated stage (maintaining the mouse body temperature at 37°C) such that the ventral side of the ear was imaged. The ear skin was kept warm and moist during the imaging period by continuous superfusion of warm Tyrode's salt solution (9.6 g/L Tyrodes salt and 12 mM $NaHCO_3$). Post-capillary venules with diameters of 20-40 μm were imaged for 1-2 h using a Leica SP8 DIVE multiphoton microscope using a 25x/1.0 IRAPO water dipping objective. Tunable 680-1300 nm infrared pulsed solid state laser (SpectraPhysics) was used at an excitation wavelength of 790 nm and detection using non-descanned and tunable PMT and HyD 4Tune(TM) detectors (AF488 detection: 496-576nm, AF647 detection: 627-707nm). Z stacks of 0.5 μm were acquired at 1 min intervals using the 8,000 Hz resonant Z-scanner mode. Typically, images were acquired

at a size of 300 × 130 × 50 μm with a voxel size of approximately 0.3 (x) x 0.3 (y) x 0.5 (z) μm. The mode and dynamics of neutrophil migration was determined by manual tracking of individual neutrophils using IMARIS image analysis software, as described above.

Remote organ vascular leakage quantification

Lung vascular leakage was quantified as described previously (Owen-Woods et al., 2020). Briefly, mice were subjected to non-invasive IR injury of the cremaster muscle as described above, and upon band removal, for some experiments, an IgG2a isotype control or anti-CXCL1 mAb (1 mg/kg) was injected i.v. Mice were subjected to either 4 h or 24 h reperfusion and two h prior to culling, were injected i.v. with red (580-605) 20 nm microspheres (0.8 μl/g body weight) and Alexa Fluor 488 conjugated anti-CD31 mAb (6 μg) to assess vascular permeability and label the vasculature, respectively. Mice were culled by cervical dislocation, exsanguinated, and a thoracotomy performed to expose the heart and lungs. The descending vena cava was clamped, and a 25G needle attached to a 10 mL syringe containing ice cold 2% PFA in PBS was inserted into the right ventricle. The pulmonary vasculature was perfusion fixed at a flow rate of 1 mL/min using a syringe pump. The lung lobes were dissected from the animal, and placed whole mount onto a coverslip, and imaged immediately using an inverted Zeiss LSM 800 (Carl Zeiss) confocal laser scanning microscope. Serial Z stacks were acquired with an oil immersion 40x/1.3 objective lens at a resolution of 0.156 × 0.156 × 0.3 μm (x, y, and z respectively). Images were reconstructed in 3D offline using IMARIS. Microsphere leakage into the alveolar space was quantified as MFI of microspheres outside of a CD31 isosurface generated using the IMARIS software tool. MFI values were quantified from 6-10 images per tissue from multiple lung lobes.

In some experiments, remote organ vascular permeability was quantified by accumulation of the plasma protein tracer Evans blue. Here, Evans blue solution 1% (w/v in PBS) was injected i.v. (5 μl/g body weight) 30 min prior to sacrifice by exsanguination followed by a whole body vascular washout using 10 mL PBS. Organs were collected and tissue accumulated dye was quantified by eluting in 300 μl of formamide for 18 h at 56°C. Optical density (OD) readings taken at 620 nm were normalized to formamide alone and used as a measure of Evans blue extravasation.

Flow cytometry

The level of bone marrow cell engraftment in chimeric mice, MC senescence and apoptosis, neutrophil, and red blood cell phenotyping were assessed by flow cytometry. When necessary, samples were incubated with ACK erythrocyte lysis buffer (150 mM NH_3Cl, 1 mM $KHCO_3$ and 1 mM EDTA) for 3 to 5 min. Samples were then washed and incubated in staining buffer (2 mM EDTA and 0.5% BSA in PBS) and then incubated with anti-CD16/CD32 antibodies (5 μg/mL) for 15 min at 4°C to block Fc receptor-mediated antibody binding. Finally, samples were stained with primary antibodies directly conjugated with appropriate fluorophores at 4°C for 30 min. Briefly, following doublet exclusion, live cell populations were gated as follows: $CD45^+$ $Ly6G^+$ $CD115^-$ (neutrophils), $CD45^+$ $CD117^+$ $FC\varepsilon R1^+$ (MC) and $Ter119^+$ (erythrocytes). Accurate cells counts were validated using 123count eBeads Counting Beads (ThermoFisher Scientific) and analyzed using a LSR Fortessa flow cytometer (BD Biosciences) and FlowJo software (TreeStar).

Tracking and phenotyping of rTEM neutrophils

Peripheral dissemination of rTEM neutrophils away from cremaster muscles was achieved by tracking the cells as previously described (Owen-Woods et al., 2020). Briefly, naive mice were injected i.v. with biotin anti-Ly6G mAb (2 μg), and 10 min later, mice were subjected to non-invasive cremasteric ischemia for 40 min via the application of two orthodontic bands to the intact testes and scrotum. Upon reperfusion, mice received a local i.s. injection of AF647-streptavidin (1 μg) for 1-4 h. Whole blood was collected in PBS containing 50 mM EDTA via the inferior vena cava, and lung vascular washout was collected as previously described (Colom et al., 2015; Woodfin et al., 2011). Briefly, pulmonary vascular neutrophils were obtained via the flushing of the pulmonary vasculature. For this purpose, the descending vena cava and the aorta were clamped, the vasculature perfused with 10 mL of wash buffer (2mM EDTA and 0.5% BSA in PBS) via the right atrium and pulmonary vascular washout was collected via the left ventricle. Samples were then prepared for analysis of neutrophil populations as described in the flow cytometry section. For some experiments, neutrophils were sorted by flow cytometry into $streptavidin^{lo}$ and $streptavidin^{hi}$ populations from the whole blood of young or aged mice subjected to IR injury as described above and these purified neutrophils (≥99%) were then injected i.v. (~2500-3800 cells per mouse) into naive aged or young WT recipients for 4 or 24 h prior to assessment of distant organ damage by quantifying local extravasation of i.v. Evans blue.

Neutrophil GRK2 protein expression analysis

Neutrophils were isolated from bone marrow of *Mrp8-Cre;Grk2*$^{fl/fl}$*;Lyz2-EGFP-ki* or *Grk2*$^{fl/fl}$*;Lyz2-EGFP-ki* mice using a neutrophil isolation kit (Miltenyi Biotec) according to the manufacturer's instructions. Pure neutrophil (> 95% as assayed by flow cytometry) and non-neutrophil populations were collected for GRK2 protein expression by western blot. Cells were lysed in 1x Laemmli Buffer, denatured at 95°C for 5 min and subjected to standard Western Blot analysis using an anti-GRK2 primary mAb (Genetex) and a horseradish peroxidase-conjugated secondary Ab (Dako). Proteins were visualized by enhanced chemiluminescence acquired on a c600 camera (Azure Biosystems).

Mast cell depletion

Depletion of cremasteric MCs was achieved through the use of an anti-c-kit mAb by adapting previous protocols (Brandt et al., 2003). Briefly, mice were injected a total of 850 μg per mouse of the anti-c-kit mAb ACK.2 or isotype control in a series of i.p. and i.s. injections over a 7-day period. Typically, on day 1, mice were injected i.p. with 250 μg mAb, followed by daily i.s. injections of 150 μg mAb on days 2–5. Experimentation and assessment of MC depletion efficiency was assessed by avidin staining and confocal microscopy of cremaster muscles on day 7.

Mast cell collection and analysis

Murine MCs were harvested by peritoneal lavage as follows: naive mice were sacrificed by cervical dislocation and 5 mL of lavage buffer (2 mM EDTA, 0.25% BSA in PBS) was injected into the peritoneal cavity and incubated for 3 min prior to collection. MCs were identified within the harvested cell suspension as described in the flow cytometry section using a LSR Fortessa flow cytometer (BD Biosciences) and FlowJo software (TreeStar) and subsequent parameters were analyzed. To quantify MC senescence, cells were incubated in suspension in OptiMEM buffer (ThermoFisher Scientific) containing 100 nM of Bafilomycin (ThermoFisher Scientific) for 1 h at 37°C in order to increase the intracellular pH. The cell suspensions were then supplemented with 33 μM of C_{12}FDG (ThermoFischer Scientific) for 2 h prior to analysis of C_{12}FDG MFI. MC apoptosis was assessed as previously described (Asai et al., 2001). Briefly, peritoneal cells were stained with fluorescently labeled primary mAbs as described in the flow cytometry section and incubated with 500 μl of Annexin V binding buffer (140 mM NaCl, 2.5 mM $CaCl_2$ and 10 mM HEPES at pH 7.4) containing 5 μl of FITC-Annexin V (BD) and 5 μl of propidium iodide (Biolegend) for 15 min. Apoptotic cells were defined as Annexin-V^+/Propidium iodide$^-$. MC granularity was assessed using the flow cytometric side scatter profile.

QUANTIFICATION AND STATISTICAL ANALYSIS

Data analysis was performed using Prism software (GraphPad). All data are expressed as mean ± SEM and exact n numbers for each dataset are detailed in the figure legends. Differences between two groups were assessed for statistical significance using two-tailed paired/unpaired Student's t tests or Fischers exact test as appropriate. One way or two-way ANOVA with Tukey, Dunnett or Holm Sidak post hoc tests were performed for multiple group comparisons as appropriate. Data were classed as statistically significant when $p < 0.05$.

Immunity, Volume *54*

Supplemental information

Age-related changes in the local milieu of inflamed tissues cause aberrant neutrophil trafficking and subsequent remote organ damage

Anna Barkaway, Loïc Rolas, Régis Joulia, Jennifer Bodkin, Tchern Lenn, Charlotte Owen-Woods, Natalia Reglero-Real, Monja Stein, Laura Vázquez-Martínez, Tamara Girbl, Robin N. Poston, Matthew Golding, Rebecca S. Saleeb, Aude Thiriot, Ulrich H. von Andrian, Johan Duchene, Mathieu-Benoit Voisin, Cleo L. Bishop, David Voehringer, Axel Roers, Antal Rot, Tim Lämmermann, and Sussan Nourshargh

Figure S1

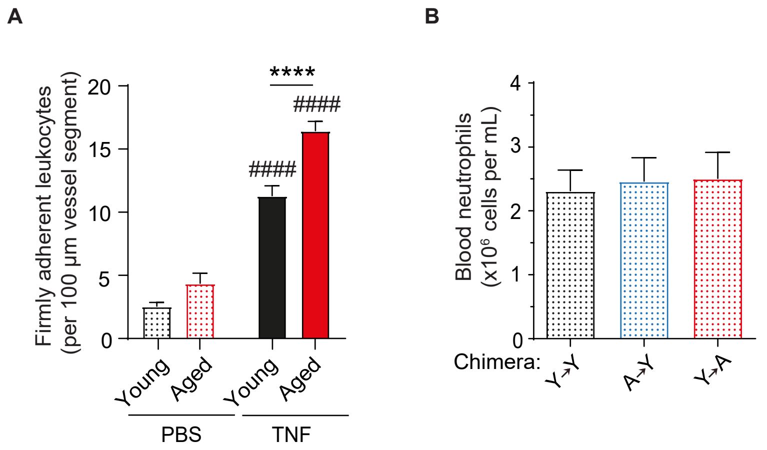

Figure S1 (Related to Figure 1). Leukocyte adhesion responses in TNF-stimulated venules of WT mice and chimeric mice characterization. (**A**) Young (2-4 months) and aged (≥18 months) mice were treated intrascrotally (i.s.) with PBS or TNF and leukocyte firm adhesion in cremasteric post-capillary venules quantified by brightfield IVM (n=3-7 mice/group). (**B**) Total neutrophil blood counts in chimeric mice (as generated in Figure 1H) at 4 weeks post irradiation and bone marrow transfer (n≥10 mice/group). Means ± SEM, ####p< 0.0001 as compared to age-matched controls, ****p<0.0001 as indicated.

Figure S2

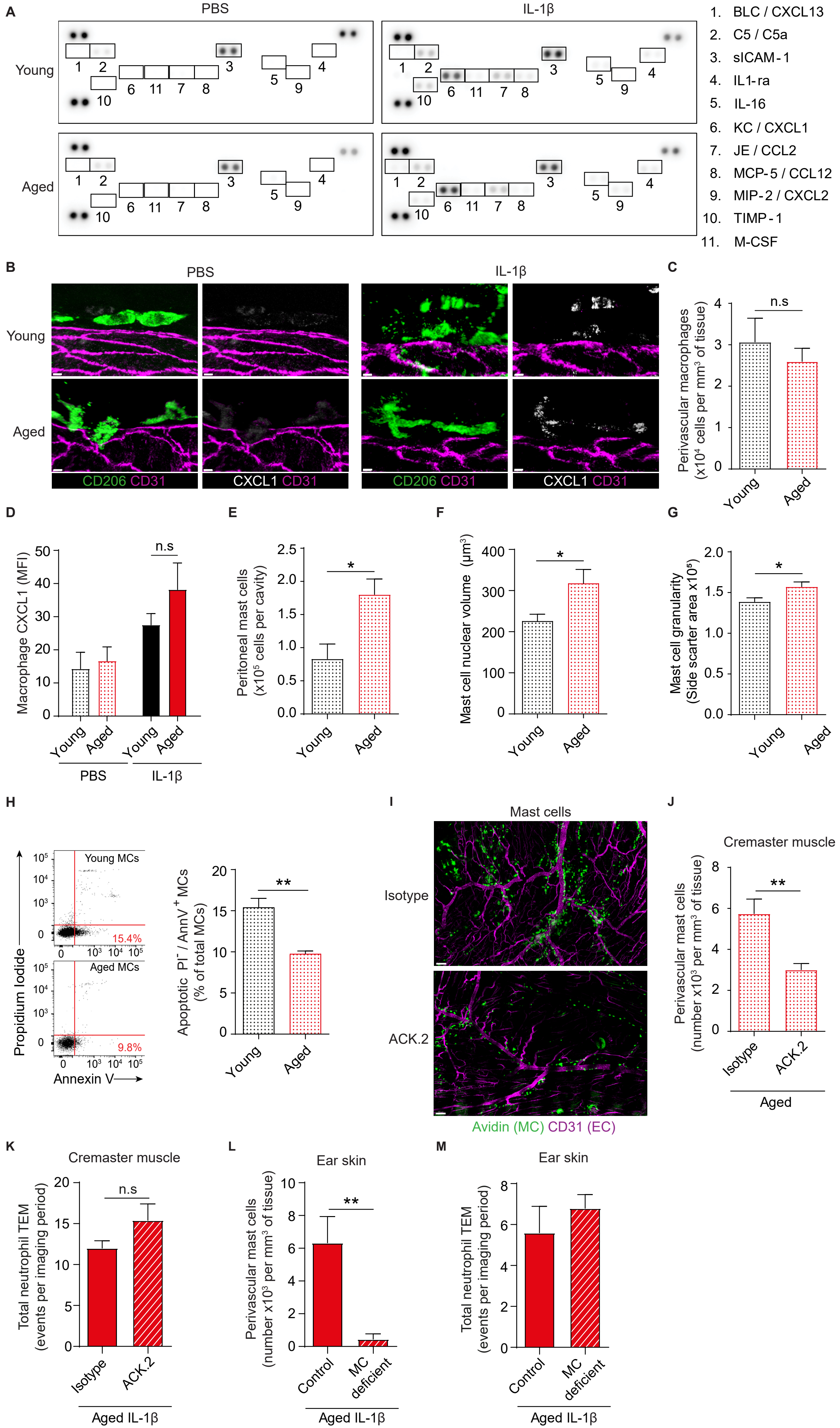
A
PBS
IL-1β
Young
Aged
1. BLC / CXCL13
2. C5 / C5a
3. sICAM-1
4. IL1-ra
5. IL-16
6. KC / CXCL1
7. JE / CCL2
8. MCP-5 / CCL12
9. MIP-2 / CXCL2
10. TIMP-1
11. M-CSF
B
PBS
IL-1β
Young
Aged
CD206 CD31
CXCL1 CD31
C
Perivascular macrophages (x10^4 cells per mm^3 of tissue)
n.s
Young
Aged
D
Macrophage CXCL1 (MFI)
n.s
Young
Aged
PBS
IL-1β
E
Peritoneal mast cells (x10^5 cells per cavity)
*
Young
Aged
F
Mast cell nuclear volume (µm^3)
*
Young
Aged
G
Mast cell granularity (Side scarter area x10^5)
*
Young
Aged
H
Young MCs
15.4%
Aged MCs
9.8%
Propidium Iodide
Annexin V
Apoptotic PI^- / AnnV^+ MCs (% of total MCs)
**
Young
Aged
I
Mast cells
Isotype
ACK.2
Avidin (MC) CD31 (EC)
J
Cremaster muscle
Perivascular mast cells (number x10^3 per mm^3 of tissue)
**
Isotype
ACK.2
Aged
K
Cremaster muscle
Total neutrophil TEM (events per imaging period)
n.s
Isotype
ACK.2
Aged IL-1β
L
Ear skin
Perivascular mast cells (number x10^3 per mm^3 of tissue)
**
Control
MC deficient
Aged IL-1β
M
Ear skin
Total neutrophil TEM (events per imaging period)
Control
MC deficient
Aged IL-1β

Figure S2 (Related to Figure 2). Characterization of pro-inflammatory mediator generation and stromal cells in young and aged stimulated cremasteric tissues. (**A**-**D**) Young and aged WT mice were stimulated i.s. with IL-1β or PBS for 4 h. (**A**) Inflammatory mediator detection by protein array in cremaster muscles (n=3 mice/condition). (**B**) Representative confocal images depicting CXCL1 expression by perivascular macrophages (CD206; scale bar: 5 μm), (**C**) macrophage numbers associated with post-capillary venules and (**D**) immunoreactive CXCL1 expression (MFI) in these cells (n=4-6 mice/group). (**E**) Number of peritoneal mast cells (MCs) in naïve young and aged WT mice as assessed by flow cytometry (n=3-5 mice/group). (**F**) Quantification of MC nuclear volume in un-stimulated ear skins as assessed by confocal microscopy (n=3 mice/group). Peritoneal MCs acquired from un-stimulated young and aged mice assayed for (**G**) granularity (n=5 mice/group) and (**H**) apoptosis (n=6-7 mice/group), both by flow cytometry. **(I-K)** Aged WT mice were treated with anti-CD117 (ACK.2) mAb or isotype control for mast cell (MC) depletion. (**I**) Representative tile scan confocal images of the cremaster muscle microcirculation (scale bar: 300 μm), (**J**) quantification of MCs (avidin) associated with post-capillary venules (CD31) (n=3-5 mice/group) and (**K**) total neutrophil TEM events in IL-1β-stimulated control or mast cell depleted aged mice. (**L**) Quantification of MC numbers associated with post-capillary venules in ear skins stained with avidin (MCs) and anti-CD31 mAb (ECs) and (**M**) total neutrophil TEM events in IL-1β-stimulated ear skin of MC deficient (*Mcpt5-Cre-R-DTA)* aged mice and their littermate controls (n=5-7 mice/group). Means ± SEM, *p<0.05, **p<0.01, n.s. not significant as indicated.

Figure S3

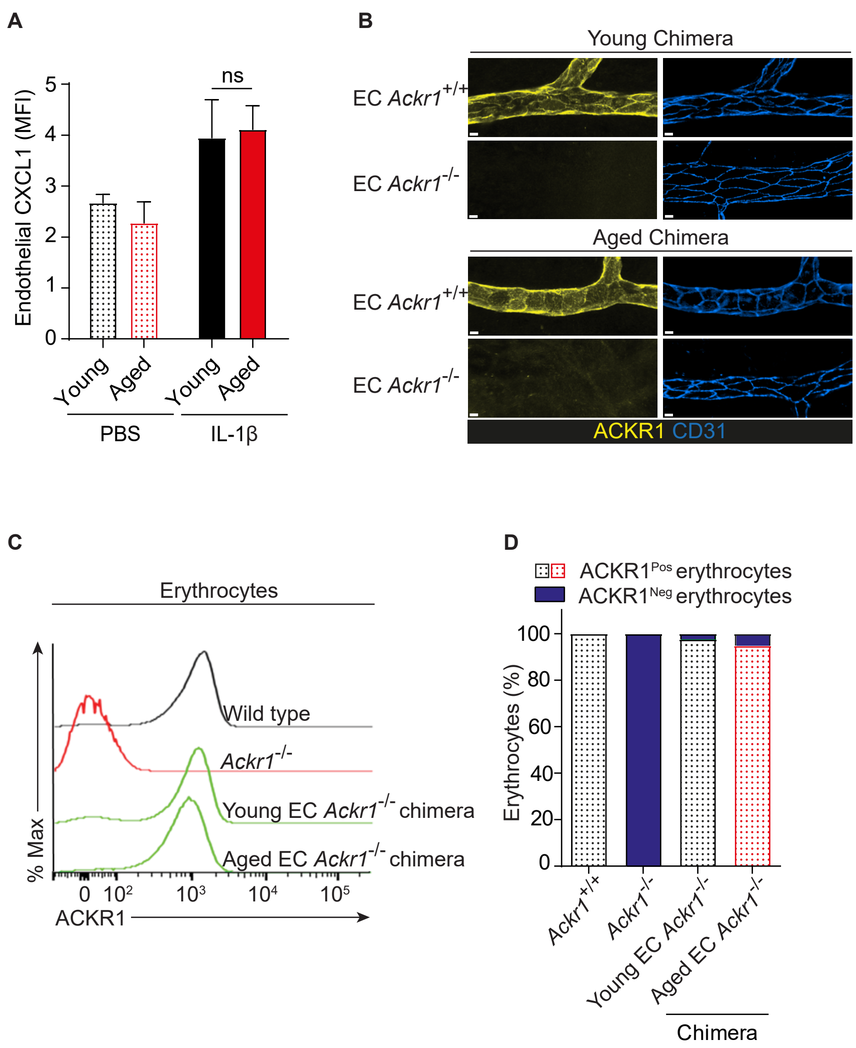

Figure S3 (Related to Figure 3) EC CXCL1 expression and EC *Ackr1*$^{-/-}$ chimeric mice characterization. (**A**) EC CXCL1 quantification (MFI) in young and aged mouse cremasteric post capillary venules (PCVs) treated with PBS or IL-1β (n = 6-7 mice/group). (**B**) Representative confocal images of ear skin PCVs immunostained for ACKR1 and CD31 from young and aged EC *Ackr1*$^{+/+}$ and EC *Ackr1*$^{-/-}$ chimeric mice (scale bar: 20 µm). (**C**) Flow cytometry analysis of erythrocyte (Ter119^{+}) ACKR1 expression and **(D)** relative percentages of ACKR1^{+} and ACKR1^{-} erythrocytes in peripheral blood of chimeric mice generated as described in Figure 3H (n=3-4 mice/group). Means ± SEM, n.s. not significant as indicated.

Figure S4

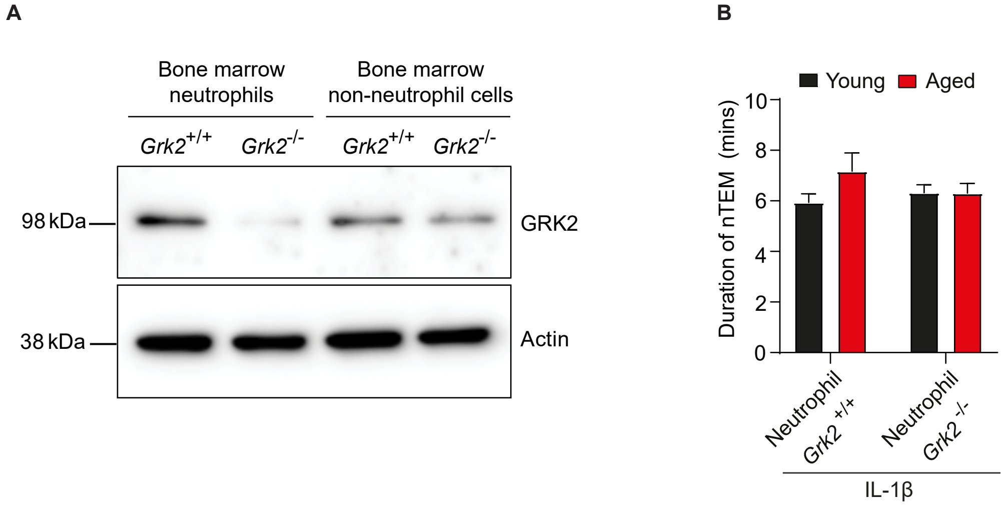

Figure S4 (Related to Figure 4). Validation of neutrophil GRK2 deficiency and analysis of neutrophil TEM duration. (**A**) GRK2 expression in purified bone marrow derived neutrophils and non-neutrophil cells of neutrophil *Grk2*$^{+/+}$ and *Grk*2$^{-/-}$ chimeric mice as analyzed by western blot (loading control: Actin). (**B**) Duration of neutrophil normal TEM (mins) as assessed by confocal IVM in IL-1β-stimulated cremaster muscles of young and aged neutrophil *Grk2*$^{+/+}$ and *Grk*2$^{-/-}$ chimeric mice (n=30-82 neutrophils analyzed/group). Means ± SEM.

Figure S5

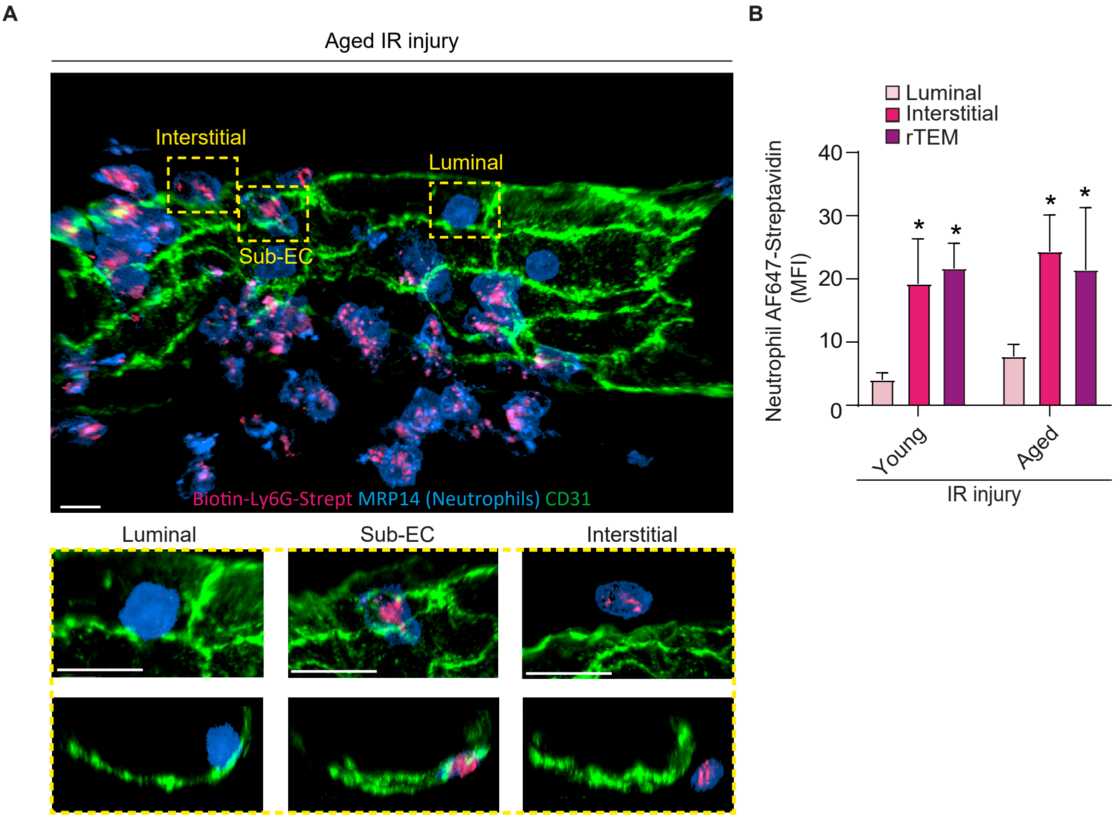

Figure S5 (Related to Figure 5). Validation of biotin-Ly6G-AF647-Streptavidin cell tracking, as a method for specifically labelling neutrophils that have breached the endothelium. Young and aged WT mice were injected i.v. with biotinylated anti-Ly6G mAb, subjected to sham or cremasteric IR injury and AF647-Strept was applied locally to cremaster muscles. (**A**) Representative confocal images of neutrophil AF647-Strept signal in the vascular lumen, subendothelial space and interstitium during IR injury in aged WT mice (dashed boxes delineate magnified areas; scale bar: 10 μm). (**B**) AF647-Strept quantification (MFI) of neutrophils within the lumen, interstitial tissue, and cells undergoing rTEM in IR-stimulated cremaster muscles of young and aged *Lyz2-EGFP-ki* mice (n=3-4 mice/group). Means ± SEM * $p<0.05$ as compared to MFI of age-matched luminal neutrophils.

Figure S6

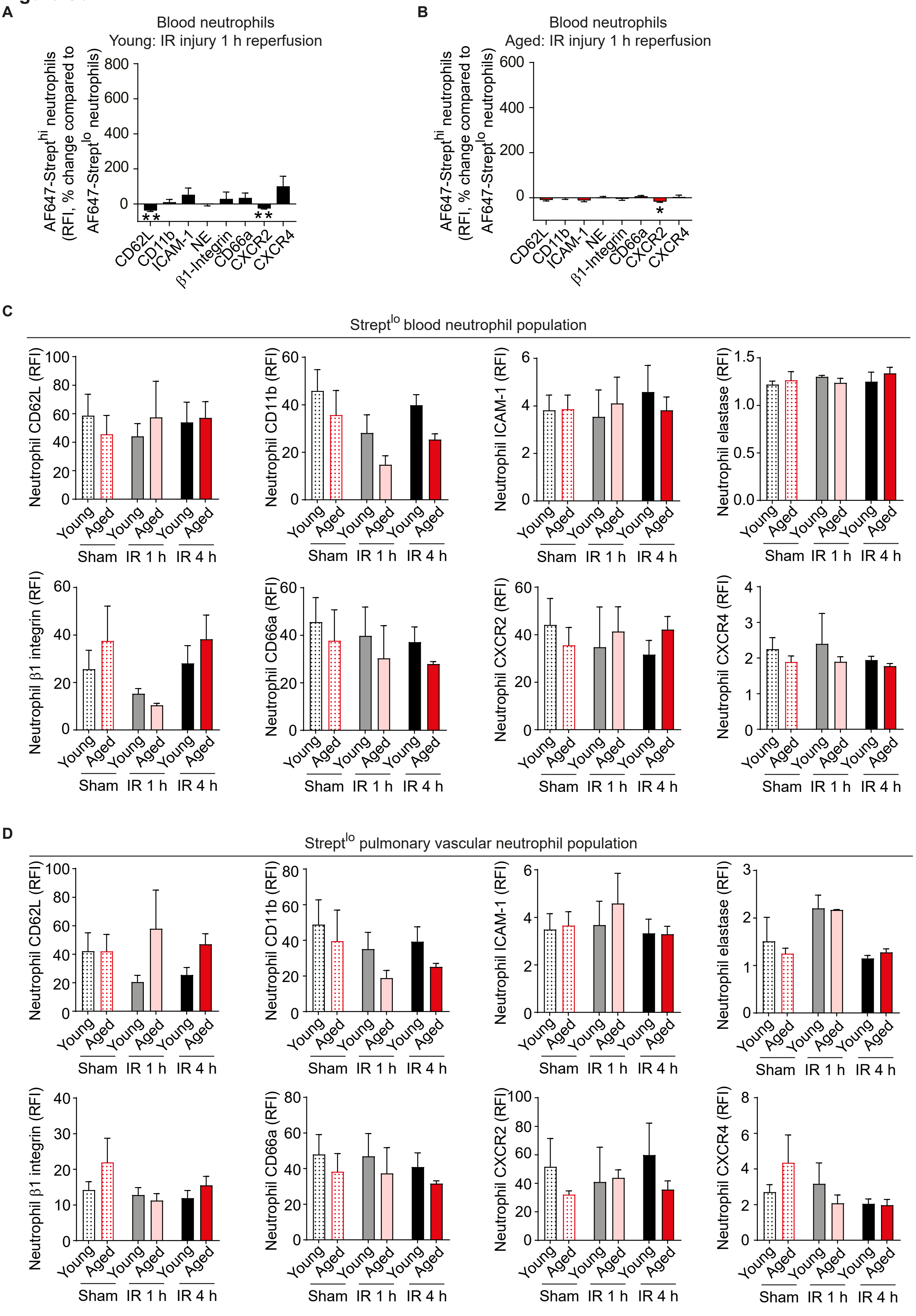
A
Blood neutrophils
Young: IR injury 1 h reperfusion
AF647-Strept^hi neutrophils (RFI, % change compared to AF647-Strept^lo neutrophils)
CD62L
CD11b
ICAM-1
NE
β1-integrin
CD66a
CXCR2
CXCR4
B
Blood neutrophils
Aged: IR injury 1 h reperfusion
C
Strept^lo blood neutrophil population
Neutrophil CD62L (RFI)
Neutrophil CD11b (RFI)
Neutrophil ICAM-1 (RFI)
Neutrophil elastase (RFI)
Neutrophil β1 integrin (RFI)
Neutrophil CD66a (RFI)
Neutrophil CXCR2 (RFI)
Neutrophil CXCR4 (RFI)
Young
Aged
Sham
IR 1 h
IR 4 h
D
Strept^lo pulmonary vascular neutrophil population

Figure S6 (Related to Figure 6). Phenotypic analysis of AF647-Strept^{lo} and AF647-Strept^{hi} blood and pulmonary vascular neutrophils. Young and aged WT mice were injected i.v. with biotinylated anti-Ly6G mAb, subjected to sham or cremasteric IR injury and AF647-Strept was applied locally to cremaster muscles. Relative expression levels of indicated markers on AF647-Strept^{hi} neutrophils relative to levels on AF647-Strept^{lo} neutrophils in the blood following 1 h reperfusion in **(A)** young and **(B)** aged IR-stimulated mice. Relative expression of indicated markers on AF647-Strept^{lo} (**C**) blood neutrophils and (**D**) neutrophils from the pulmonary vasculature (n=5 mice/group). Means ± SEM *p<0.05, **p<0.01 as compared to AF647-Strept^{lo} neutrophils of the same group.

Figure S7

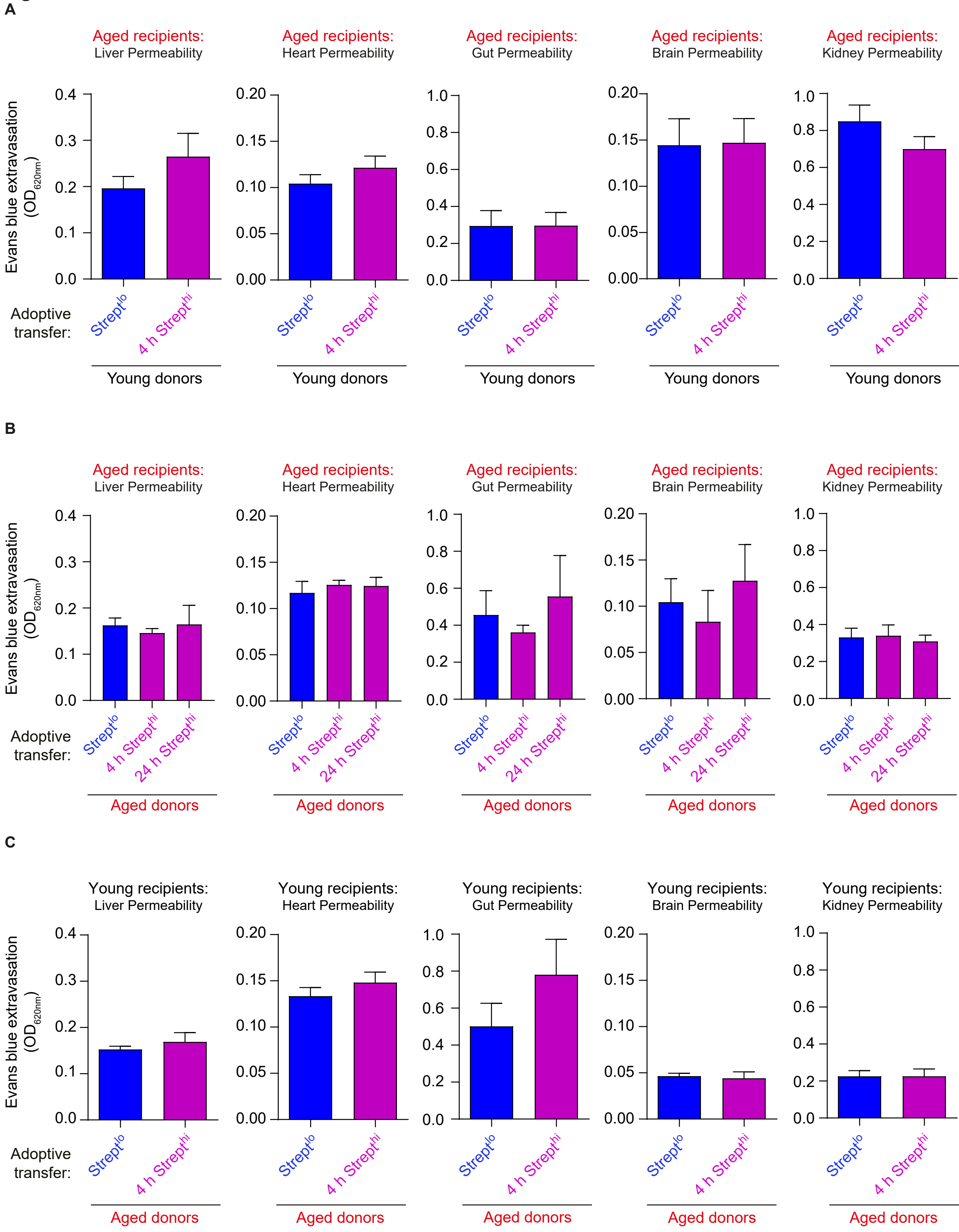

Figure S7 (Related to Figure 6). Impact of AF647-Strept^{lo} and AF647-Strept^{hi} neutrophils on organ permeability. Extravasation of i.v. Evans blue in multiple tissues of (**A**) aged recipients 4 h post i.v. injection of neutrophils sorted from young donors, (**B**) aged recipients 4 or 24 h post i.v. injection of neutrophils sorted from aged donors, and (**C**) young recipients 4 h post i.v. injection of neutrophils sorted from aged donors (n=4-7 mice/group). Means ± SEM.
